# Novel function of THEMIS2 in the enhancement of cancer stemness and chemoresistance by releasing PTP1B from MET

**DOI:** 10.1038/s41388-021-02136-2

**Published:** 2022-01-01

**Authors:** Wei-Chieh Huang, Jia-Hau Yen, Yu-Wen Sung, Shiao-Lin Tung, Po-Ming Chen, Pei-Yi Chu, Ya-Chi Shih, Hsiang-Cheng Chi, Yi-Ching Huang, Shih-Jei Huang, Lu‐Hai Wang

**Affiliations:** 1 Graduate Institute of Integrated Medicine, China Medical University, Taichung, Taiwan; 2 Chinese Medicine Research Center, China Medical University, Taichung, Taiwan; 3 Research Cancer Center for Traditional Chinese Medicine, Department of Medical Research, China Medical University Hospital, Taichung, Taiwan; 4 Department of Obstetrics and Gynecologics, China Medical University Hospital, Taichung City, Taiwan; 5 Department of Hematology and Oncology, Ton-Yen General Hospital, Hsinchu County, Taiwan; 6 Department of Nursing, Hsin Sheng Junior College of Medical Care and Management, Taoyuan City, Taiwan; 7 Department of Pathology, Show Chwan Memorial Hospital, Chung-Shang Road, Changhua County, Taiwan

**Keywords:** Breast cancer, Cancer stem cells, Cancer therapeutic resistance

## Abstract

Triple negative breast cancer (TNBC) possesses poor prognosis mainly due to lack of effective endocrine or targeted therapies, aggressive nature and high rate of chemoresistance. Cancer stem cells (CSCs) are considered to play critical roles in cancer recurrence and chemoresistance. THEMIS2 was identified as the sole common elevated gene in three triple negative breast cancer (TNBC) and two ovarian CSC lines. We discovered an intrinsic signaling scaffold function of THEMIS2, which acts as a novel regulator of cancer stemness in promoting multiple cancer stemness properties including sphere formation, stemness markers expression, chemoresistance and tumorigenicity with low numbers of cancer cells implantation. For the first time, we demonstrated that THEMIS2 specifically enhanced MET activating phosphorylation by suppressing the association of protein-tyrosine phosphatases 1B (PTP1B) with p-MET and MET, which accounted mainly for THEMIS2-mediated effect on cancer stemness and chemoresistance. Increased THEMIS2 expression was associated with *poor survival* in TNBC patients and in patients from our breast cancer cohort. We found that non-cytotoxic dosages of cryptotanshinone (CPT) could potently inhibit cancer stemness, chemoresistance and tumorigenicity by suppressing expression of THEMIS2. Notably, stable overexpression of THEMIS2 is associated with enhanced sensitivity toward Capmatinib and CPT treatment. Expression levels of THEMIS2 and p-MET protein were positively correlated in the 465 breast cancer specimens. Our study revealed the novel oncogenic role of THEMIS2 and its underlying mechanism via suppressing PTP1B association with MET and thus leading to its activation. Our findings suggest that THEMIS2 could be a biomarker for MET targeted therapy and also provide a potential clinical application using low dosages of CPT for treatment of THEMIS2 positive TNBC.

## Introduction

Breast cancer is the second leading causes of female cancer death [1]. Triple-negative breast cancer (TNBC) is associated with poor prognosis because of the lack of effective endocrine or targeted therapies, its aggressive and recurrent nature, and its resistance to chemoresistance [2]. The main treatment modalities for TNBC include surgery, radiotherapy and chemotherapy [3]. The few targeted therapies including inhibitors of mammalian target of rapamycin (mTOR), epidermal growth factor receptor (EGFR), fibroblast growth factor receptor 2 (FGFR2) and vascular endothelial growth factor (VEGF) have been developed recently [3]. Due to the high rate of recurrence and chemoresistance associated with TNBC [2], the development of new therapeutic agents is urgently required.

Thymocyte expressed molecule involved in selection 2 (THEMIS2), also known as ICB-1 or C1orf38 has been shown as a differentiation-associated gene and is expressed primarily in B cells and macrophages [4, 5]. Furthermore, it can lower the threshold for B cell activation during positive selection and plays a pivotal role in controlling macrophage toll-like receptor responses [4, 6]. The transcription of THEMIS2 is estrogen-responsive and the knockdown of THEMIS2 enhances estrogen responsiveness in both breast and ovarian cancer cells [5, 7]. Although THEMIS2 plays a role in inflammation, its specific actions and mechanism in regulating cancer stemness, metastasis, and recurrence has never been studied.

MET is a promising target in cancer therapy because its signaling axis is involved in several types of cancer [8]. One study reported that MET is overexpressed in approximately 52% of TNBCs cases [9]. Hepatocyte growth factor (HGF)/ MET signaling is known to be able to initiate the epithelial-mesenchymal transition (EMT), of which the association with cancer stem cell (CSC) generation has been established [10]. CSCs are a very small subpopulation of tumor cells shown to be involved in tumor initiation, recurrence, metastatic potential, and chemoresistance [11–13]. Recent studies have indicated that CSCs also play a crucial role in chemoresistance and recurrence in TNBC [2, 14]. Although several small molecule MET inhibitors such as crizotinib, capmatinib and cabozantinib have been used in the treatment of non-small cell lung cancer (NSCLC) and hepatocellular carcinoma (HCC) [15, 16], only a few MET inhibitors such as cabozantinib and CPDA showed modest activity or preclinical efficacy in breast cancer and TNBC [15]. The role of MET in the CSCs of TNBC has yet to be delineated.

In this study, we successfully enriched CSC lines from three established human TNBC and two ovarian cancer lines and identified THEMIS2 to be the sole common gene with increased expression in the five CSCs enriched lines. THEMIS2 could potently promote in vitro and in vivo stemness properties of TNBC. We also discovered a novel mechanism with regard to the regulatory function of THEMIS2 in enhancing MET activation phosphorylation to regulate cancer stemness by suppressing the association of PTP1B with p-MET. This is the first report revealing the inhibitory effect of cryptotanshinone (CPT), a powerful STAT3/STAT5 inhibitor [17], on THEMIS2–MET axis leading to inhibition of cancer stemness and lung metastasis. Furthermore, expression of THEMIS2 correlated with poor clinical outcomes in TNBC patients and breast cancer patients. Of interest, elevated levels of THEMIS2 have been positively associated with response to MET inhibitors. In short, we discovered that THEMIS2 controls cancer stemness and chemoresistance mainly by regulating PTP1B–p-MET interaction and MET activation. Overexpression of THEMIS2 increased TNBC sensitivity to MET inhibitors Capmatinib and CPT, suggesting that THEMIS2 could provide new therapeutic implications.

## Results

### Establishment of sphere-derived CSC-enriched TNBC and ovarian cancer lines and the identification of their differentially expressed genes

**Fig. 1:**
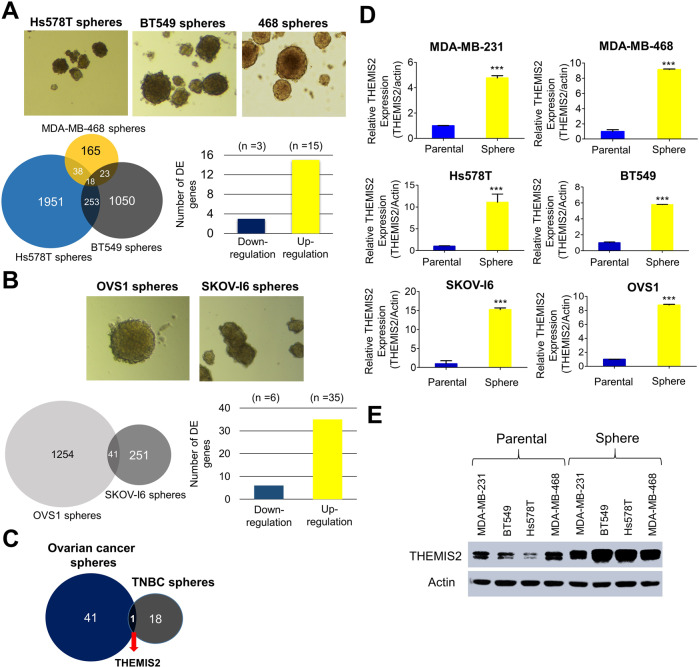
Identification of THEMIS2 as the sole common differentially expressed (DE) gene among the sphere-derived CSC-enriched cell lines for TNBC and ovarian cancer. **A** Top: Formation of spheres under the stem-cell-selective condition on day 8 after culturing from parental Hs578T, BT549, and MDA-MB-468 cells; bottom: the number of DE genes implicated in Hs578T, BT549, and MDA-MB-468 sphere cells are shown in the Venn diagram. Downregulated and upregulated DE genes (indicated in blue and yellow, respectively) are presented in the bar chart. **B** Top: Formation of spheres under a similar condition from parental OVS1 and SKOV-I6 lines; bottom: the number of DE genes implicated in OVS1 and SKOV-I6 sphere cells is shown in the Venn diagram. **C** The DE genes implicated in TNBC and ovarian cancer sphere cells were compared to identify a sole common DE gene as shown in the Venn diagram. **D** The mRNA expression levels of THEMIS2 in parental MDA-MB-231, MDA-MB-468, Hs578T, BT549, SKOV-I6, and OVS1 cells, as well as in their respective sphere cells, were analyzed using qRT-PCR. **E** The protein expression levels of THEMIS2 in TNBC sphere cells relative to those of their parental cells were analyzed by immunoblotting. Actin served as an internal control. The histograms represent means ± standard errors (SEs) from three independent experiments (***P < 0.001).

To address the problem of drug resistance and recurrence in malignant tumors, we used cell lines from TNBC and ovarian cancer, which are known for their high chemoresistance and recurrence tendencies, for the enrichment of CSCs and the identification of relevant genes as potential therapeutic targets [18, 19]. We enriched the CSCs of the TNBC (Hs578T, BT549, and MDA-MB-468) and ovarian cancer (SKOV-I6 and OVS1) lines by growing them in stem-cell-selective conditions conducive to sphere formation as described in the Methods section. The floating spheres possessed a rounded, confluent three-dimensional configuration and in most cases reached 50–100 μM in diameter after 5–8 days in culture (Fig. 1A, *top*). To identify the CSCs’ differentially expressed (DE) genes and signaling pathways relevant to cancer stemness, we performed RNA sequencing by using the Partek software package and Gene Ontology (GO) software to compare parental TNBC cells, and their corresponding sphere-derived cells with regard to gene expression profiles. A total of 18 DE genes were identified in the intersection area of the three TNBC pairs, as shown in the Venn diagram in Fig. 1A (*bottom*). Relative to their corresponding parental cells, 3 and 15 of the 18 DE genes were downregulated and upregulated in the sphere cells, respectively (Fig. 1A, *bottom* and Table 1). Similarly, we enriched CSCs from the OVS1 and SKOV-I6 ovarian cancer cell lines by using the sphere-formation method (Fig. 1B, *top*). 41 DE genes expressed in the sphere cells in the intersection area of the two pairs were identified as shown in the Venn diagram in Fig. 1B (*bottom*). Among those 41 genes, 6 and 35 were downregulated and upregulated, respectively (Fig. 1B, *bottom* and Table 1). THEMIS2 was the only gene that was commonly expressed among the 18 and 41 DE genes as shown in the Venn diagram in Fig. 1C. The increased THEMIS2 mRNA expression (Fig. 1D) and protein level (Fig. 1E) in the sphere cells was confirmed through qRT-PCR and immunoblotting.

**Table 1:** The genes of differential expression in TNBC and ovarian cancer sphere cells.

The 18 genes of differential expression in TNBC sphere cells				
Downregulation	Upregulation			
MYPN	NDRG1	44078	FN1	APOE
IL7R	BST2	THEMIS2	ADAMTS7	SERPING1
ALDH1A3	AKR1C2	OLFML2B	TMC4	RP11-443P15.2
	FIBIN	ALOX15B	GSTM2	
The 41 genes of differential expression in ovarian cancer sphere cells				
Downregulation	Upregulation			
ADAMTS15	ACSS2	HMGCS1	PPFIA4	
CYP24A1	AKAP9	IDI1	SCD	
GDF6	ALDOC	IL1B	SH2D3C	
TERT	ANKRD12	INSIG1	SLC2A3	
THBD	ANKRD33	LEMD1	SNCA	
THBS1	CD14	LINC00313	SQLE	
	CEP290	LIPG	STARD4	
	CTHRC1	LUCAT1	STC1	
	CXCR4	MLPH	SYTL2	
	EGR3	MSMO1	THEMIS2	
	GOLGA4	PCSK9	WFDC3	
	GPNMB	PLAT		

### THEMIS2 regulated cell migration/invasion, colony formation, cell proliferation and cancer stemness properties

**Fig. 2:**
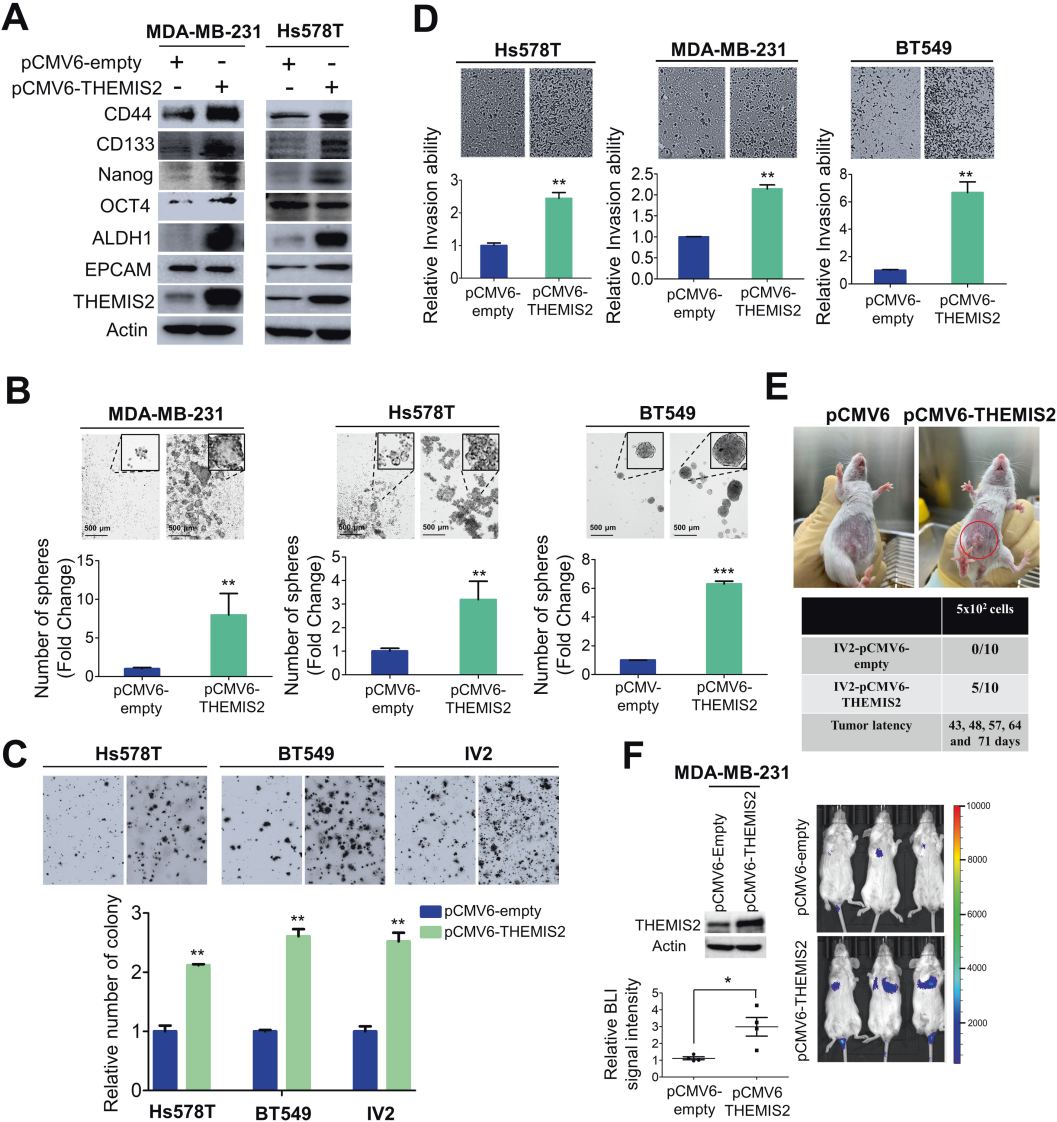
THEMIS2 was able to promote cancer stemness properties in TNBC cells. **A** The protein expression levels of CSC markers in THEMIS2-overexpressing MDA-MB-231 and Hs578T cells, relative to those of their parental cells, were analyzed through immunoblotting. Actin was used as an internal control. **B** The sphere-forming abilities of three stable THEMIS2-overexpressing TNBC lines (MDA-MB-231, Hs578T, and BT549) relative to those of their parental cells. The histograms represent means ± SEs from three independent experiments (**P < 0.01; ***P < 0.001). **C** The colony-forming abilities of three stable THEMIS2-overexpressing TNBC lines (Hs578T, BT549, and MDA-MB-231-IV2 (IV2)) in soft agar, relative to those of their parental cells, were analyzed through a soft agar colony formation assay. The histograms represent means ± SEs from three independent experiments (**P < 0.01). **D** The invasion abilities of three stable THEMIS2-overexpressing TNBC lines (Hs578T, MDA-MB-231, and BT549), relative to those of their parental cells, were analyzed using a chemotactic cell invasion assay. The histograms represent means ± SEs from three independent experiments (**P < 0.01). **E** 5 × 10^2^ stable MDA-MB-231-pCMV6-THEMIS2 cells or stable pCMV6-control cells were orthotopically implanted into the mammary fat pads of 10 CB17-SCID mice (*n* = 10 for each group). Representative images of tumor growth and tumor latency are shown. **F** Left: THEMIS2 protein expression levels in stable THEMIS2-overexpressing MDA-MB-231 cells, as determined by immunoblotting analysis. Actin served as an internal control. The BLI image scatter plots represent means ± SEs from three independent experiments (*P < 0.05). Right: representative images of pulmonary metastasis induced by 5 × 10^5^ MDA-MB-231 cells with or without THEMIS2 overexpression in the CB17-SCID mice, 21 days after implantation (*n* = 3 for each group). Tumor growth was monitored through bioluminescent imaging. The actin bands in A served as the representative loading control of multiple immunoblots from the same batch of cell lysates. They served as the internal controls for comparing the intensity of the proteins bands stained by a given antibody.

We focused our study on THEMIS2 in TNBC to explore potential CSC-related targets for therapeutic development. We also examined the potential roles of THEMIS2 in CSCs-related properties. Overexpression of THEMIS2 in MDA-MB-231 and Hs578T cells by transfection with pCMV6-THEMIS2 to derive stable TNBC lines resulted in elevation in protein levels of the CSC markers, including CD44, CD133, Nanog, OCT4, aldehyde dehydrogenase 1 (ALDH1), and epithelial cell adhesion molecule (EPCAM), relative to those in control cells (Fig. 2A) [20, 21]. By contrast, THEMIS2 knockdown led to significant reductions in the protein levels of those CSC markers in the TNBC cells (Supplementary Fig. 1A). We also performed immunofluorescence staining to assess the levels of CD133 and ALDH1 in stable THEMIS2-overexpressing MDA-MB-231 sphere cells relative to those in their parental cells. CD133 and ALDH1 were expressed in both parental control and stable THEMIS2-overexpressing MDA-MB-231 sphere cells, and considerable augmentation of CD133 and ALDH1 expression was noted in the THEMIS2-overexpressing cells (Supplementary Fig. 1B).

THEMIS2 overexpression promoted sphere formation in TNBC cells (Fig. 2B), whereas THEMIS2 knockdown consistently inhibited it (Supplementary Fig. 2A). The clonogenic assay in soft agar, performed to assess the effect of THEMIS2 on anchorage-independent cell survival and growth, revealed that all three stable THEMIS2-expressing TNBC lines displayed greater colony forming abilities than their parental cells (Fig. 2C). Increased migration and invasion abilities were also noted in these three lines (Supplementary Fig. 2B and Fig. 2D). We further employed MTT assay and sphere formation assay to evaluate whether THEMIS2 could affect cancer cell proliferation and sphere forming abilities. Knockdown of THEMIS2 inhibited whereas ectopic expression of THEMIS2 promoted cell proliferation in MDA-MB-231 cells (Supplementary Fig. 3A), similar results were also found in sphere formation assay (Supplementary Fig. 3B). CSCs are characterized by an ability to form tumors with a relatively small number of cells. In an orthotopic xenograft assay of tumorigenicity, tumor growth was not detected in mice implanted with 500 control plasmid-transfected IV2 cells. However, tumor growth was observed in 5 out of the 10 mice implanted with IV2 cells transfected with the THEMIS2-expressing plasmid with tumor latencies of 43, 48, 57, 64, and 71 days respectively (Fig. 2E). Consistent with the promotion of cancer cell migration/invasion by THEMIS2, overexpression of THEMIS2 in MDA-MB-231 cells greatly increased their lung metastatic ability (Fig. 2F). These results suggest that THEMIS2 plays a pivotal role in regulating cancer stemness properties, including tumorigenicity and metastasis, in TNBC cells.

### THEMIS2 constitutively interacted with MET and promoted its activation

**Fig. 3:**
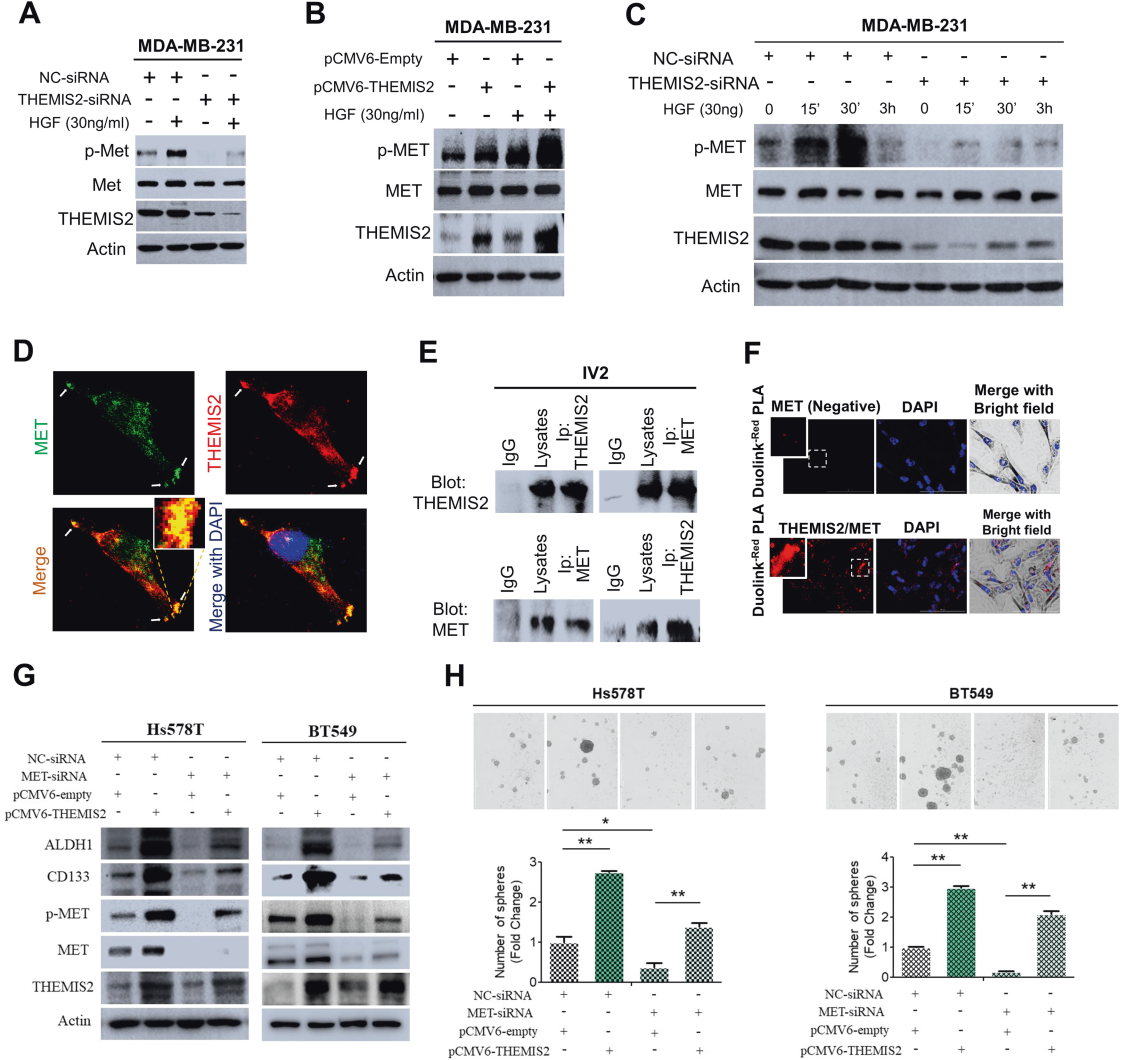
THEMIS2 was associated with MET and promoted its phosphorylation, and MET was required for THEMIS2-mediated sphere formation and CSC marker expression. **A** The protein levels of p-MET, MET, and THEMIS2 in MDA-MB-231 cells transfected with control or THEMIS2 siRNA and with or without HGF (30 ng/mL) stimulation were analyzed by immunoblotting. Actin was used as the internal control. **B** The protein levels of p-MET, MET, and THEMIS2 in MDA-MB-231cells transfected with control or pCMV6-THEMIS2 vector and with or without HGF (30 ng/mL) stimulation were similarly analyzed. Actin was used as an internal control. **C** MDA-MB-231 cells were transfected with control or THEMIS2 siRNA for 8 h, serum starved for 24 h, and then stimulated with 30 ng/mL HGF for the indicated times. **D** Immunofluorescence staining images of MET (green) and THEMIS2 (red) as indicated by the arrows, shown under confocal microscopy. Nuclei were counterstained with DAPI (blue). **E** MDA-MB-231-IV2 (IV2) cells lysates were immunoblotted directly or subjected to IP with the indicated antibodies first, followed by blotting with the indicated antibodies. IgG served as the negative control. **F** Protein–protein interactions between THEMIS2 and MET using Duolink PLA. The close proximity (<40 nm) of the two proteins is indicated by red fluorescent dots. Nuclei were counterstained with DAPI (blue). **G** Protein expression levels of ALDH1, CD133 p-MET, MET and THEMIS2 in Hs578T and BT549 cells transfected with MET-siRNAs or the indicated plasmids were analyzed by immunoblotting. Actin was used as the internal control. **H** Ectopic expression of THEMIS2 reversed the inhibition of the sphere-forming abilities due to MET knockdown. The histograms represent means ± SEs from three independent experiments (*P < 0.05; **P < 0.01).

The relevance of THEMIS2 expression in the properties of CSCs raised the possibility that high THEMIS2 expression may play a role in chemoresistance. A recent study reported that MET contributes crucially to drug resistance in a spheroid model of pancreatic cancer [22]. Several studies have suggested that MET is a new marker for CSCs [23, 24]. However, its specific link to breast CSCs remains unclear. Our results indicated that inhibiting THEMIS2 expression reduced specifically the activation of MET among the four receptor tyrosine kinases (RTKs) analyzed (Supplementary Fig. 4A). Therefore, we investigated the potential role of THEMIS2 in MET signaling. Knockdown of THEMIS2 significantly suppressed both constitutive and HGF-induced phosphorylation of MET in the MDA-MB-231 (Fig. 3A) and Hs578T cells (Supplementary Fig. 4B), whereas overexpression of THEMIS2 significantly increased both constitutive and HGF-induced phosphorylation of MET (Fig. 3B and Supplementary Fig. 4C). To further assess the effect of THEMIS2 on MET activation, siRNAs were used to deplete it in MDA-MB-231 cells, which were subsequently stimulated with HGF, as can be seen in Fig. 3C, MET phosphorylation was greatly inhibited at various time points after HGF stimulation in the THEMIS2-depleted cells. To investigate the possibility of an association between THEMIS2 and MET, we examined the localization of the two molecules in MDA-MB-231 cells using immunofluorescence. In a confocal microscopic examination, THEMIS2 and MET displayed a partial spatial colocalization pattern, especially on the surface membranes (Fig. 3D). The reciprocal coimmunoprecipitation (co-IP) assays confirmed the association between the two proteins in the MDA-MB-231-IV2 (IV2) cells (Fig. 3E). We further used Duolink PLA to detect protein–protein interactions between THEMIS2 and MET. The detected red fluorescent dots indicated that the two molecules were in close proximity (<40 nm) (Fig. 3F), and the association results were confirmed by aforementioned independent colocalization and co-IP assays. We subsequently explored the interaction between THEMIS2 and MET in sphere cells compared with their parental cells. In Duolink PLA assay, strong red signals found in clusters of Hs578T sphere cells indicating close proximity interaction between MET and THEMIS2 (Supplementary Fig. 5A). In order to compare the interaction between THEMIS2 and MET in individual sphere cells with that in their parental cells, we digested the spheres by trypsin and seeded the individual cells on slides to perform Duolink PLA assay (Supplementary Fig. 5B). Our data revealed that stronger interaction between THEMIS2 and MET was found in sphere cells when compared with their parental cells (Supplementary Fig. 5B). Similar results were obtained in co-IP assay in the three TNBC lines (Supplementary Fig. 5C).

To examine how THEMIS2 affected the MET signaling pathway, we performed immunoblotting analysis. THEMIS2 depletion led to reduced activation of phosphorylation in the downstream signaling molecules SRC, ERK, and AKT, but not c-Jun N-terminal kinase and phosphoinositide 3-kinase (PI3K), under HGF stimulation (Supplementary Fig. 4D). Notably, depletion of THEMIS2 led to reduction in the phosphorylation activation of STAT3 Tyr705, but not that of Ser727. STAT3 is activated through phosphorylation at its Tyr705 residue by RTKs, and STAT3 Tyr705 phosphorylation is critical for its dimerization and unclear translocation [25]. The protein levels of none of the above mentioned signaling molecules were affected by THEMIS2 depletion. Furthermore, the overexpression of THEMIS2-mediated MET activation and increased expression of the CSC markers, CD133 and ALDH1, in MDA-MB-231 and Hs578T cells were significantly suppressed by the MET inhibitor Capmatinib (10 μM) (Supplementary Fig. 6A). Ectopic expression of THEMIS2 partially rescued the Capmatinib -mediated inhibition of sphere formation (Supplementary Fig. 6B). A previous study indicated that treatment of Capmatinib over a period (e.g., 24 h) led to a dose-dependent increase of HER3 protein even though Capamtinib had the ability to suppress phosphorylation of EGFR and HER3 effectively in MET amplified H1993 lung cancer cells [26]. In our study, we found that treatment with Capmatinib (100 nM) over 24 hours in THEMIS2-overexpressing MDA-MB-231 and Hs578T cells resulted in significantly decreased expression of phosphorylated MET and CSC markers CD133 and ALDH1 (Supplementary Fig. 7). Although mild increase of HER3 expression was observed in THEMIS2-overexpressing MDA-MB-231 cells, but not in Hs578T cells, after treatment with Capmatinib (100 nM) over 24 hours, the expression of neither p-HER3 nor THEMIS2 was affected (Supplementary Fig. 7). In addition, transfection with MET-siRNA resulted in decreased protein expression of THEMIS2 and CSC markers including ALDH1 and CD133 in Hs578T and BT549 cells (Fig. 3G). Increased expression of phosphorylated MET as well as CSC markers were found in THEMIS2-overexpressing Hs578T and BT549 cells, whereas transfection with MET-siRNA significantly suppressed THEMIS2-mediated augmentation effect (Fig. 3G). Similar results were obtained in sphere forming assay (Fig. 3H). The above observations indicate that THEMIS2 constitutively associates with MET and regulates its activation of phosphorylation, which in part accounts for its regulation of the properties of CSCs in TNBC.

### THEMIS2 enhanced MET phosphorylation by perturbing association of PTP1B with p-MET

A study reported that PTPs function as either negative or positive regulators of signaling pathways triggered by RTKs [27]. Several reports have noted that PTP1B acts as a negative regulator in HGF-stimulated MET activation [28, 29]. To clarify the regulatory roles of PTPs on MET phosphorylation in TNBC cells, MDA-MB-231 cells were transfected with PTP1B siRNA, SHP1-siRNA or SHP2-siRNA and examined. Depletion of PTP1B, but much less so by SHP1 or SHP2 depletion, significantly increased MET phosphorylation (Supplementary Fig. 8). This indicated that PTP1B played a major role in regulating MET phosphorylation in TNBC cells. We then assessed whether depletion of PTP1B could reverse the THEMIS2 depletion–mediated inhibition of MET phosphorylation. Knockdown of PTP1B significantly rescued constitutive and HGF-induced phosphorylation of MET in MDA-MB-231 cells under THEMIS2 knockdown (Supplementary Fig. 9). This result supports the premise of the THEMIS2–PTP1B connection in the regulation of MET phosphorylation.

**Fig. 4:**
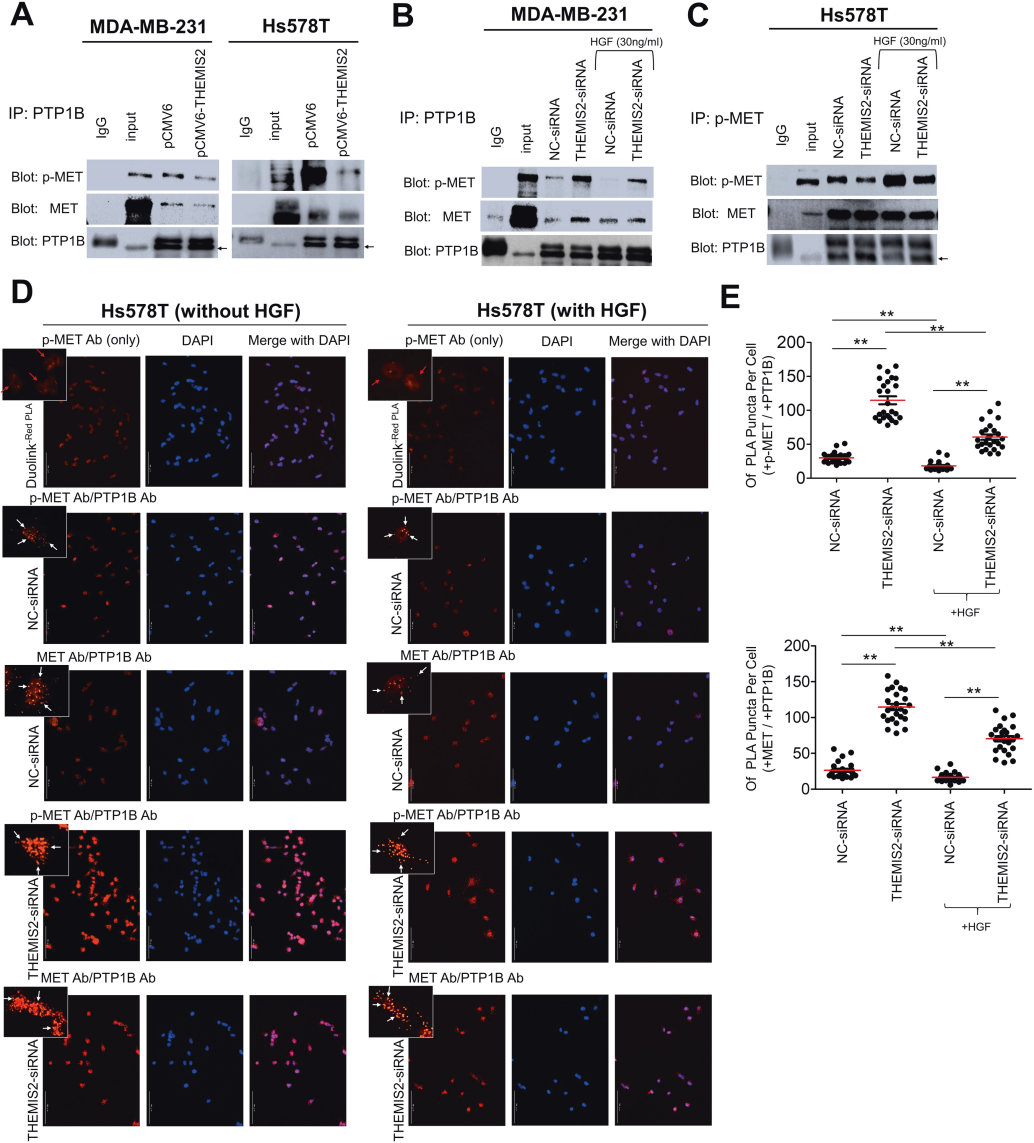
THEMIS2 regulated the interaction between p-MET and PTP1B in TNBC cells. **A** MDA-MB-231 and Hs578T cells were used in the co-IP experiments. Cell lysates were blotted directly or subjected to IP with a PTP1B antibody; this was followed by blotting with the indicated antibodies. IgG served as the negative control. **B** Cells were treated with or without HGF (30 ng) for 30 min, after which the lysates were blotted directly or subjected to IP with a PTP1B antibody first. This was followed by blotting with the indicated antibodies. IgG served as the negative control. **C** Cells were treated with or without HGF (30 ng) for 30 min, after which the lysates were blotted directly or subjected to IP with a p-MET antibody first. This was followed by blotting with the indicated antibodies. IgG served as the negative control. **D** Hs578T cells were transfected with the negative control siRNA or THEMIS2 siRNA for 8 h, serum starved for 24 h, and then treated without or with 30 ng/mL HGF for 30 min (left and right panels, respectively). A Duolink PLA was then performed. Top: p-MET antibody only served as the negative control; middle: protein–protein interactions between p-MET and PTP1B or between MET and PTP1B was analyzed using both antibodies; bottom: protein–protein interactions between p-MET and PTP1B or MET and PTP1B in THEMIS2-depleted cells. The signals are represented by *white* arrows and the nonspecific signals by red arrows. The close proximity (<40 nm) association of the two proteins in each set is indicated by small, distinct red dots, which were detected using fluorescence microscopy. Nuclei were counterstained with DAPI (blue). **E** Quantification of signals by number of PLA puncta per cell. The histograms represent means ± SEs from three independent experiments (**P < 0.01).

Because THEMIS2 significantly promoted MET phosphorylation in TNBC cells (Fig. 3 and Supplementary Fig. 4) and on the basis of the effect of PTP1B we observed, we investigated whether THEMIS2 regulated MET phosphorylation through PTP1B. We found that overexpression of THEMIS2 reduced the association between PTP1B and p-MET in four TNBC (MDA-MB-231, Hs578T, MDA-MB-468 and BT549) lines by co-IP assay (Fig. 4A, Supplementary Fig. 10). Upregulation of THEMIS2 was also found to inhibit the association of PTP1B with MET although the inhibitory effect was only modest in Hs578T cells (Fig. 4A, Supplementary Fig. 10). To support the above observations, we performed in situ Duolink PLA analysis and quantified PLA puncta to assess the impact of THEMIS2 on p-MET-PTP1B and MET-PTP1B association. Duolink PLA analysis showed similar results that overexpression of THEMIS2 reduced the interaction between MET-PTP1B and p-MET-PTP1B in MDA-MB-231 cells (Supplementary Fig. 11). Conversely, depletion of THEMIS2 significantly increased the association between PTP1B and p-MET, and also mildly increased the association between PTP1B and unphosphorylated MET in MDA-MB-231 cells with or without HGF treatment (Fig. 4B). This was confirmed by reciprocal co-IP using p-MET antibody that depletion of THEMIS2 increased the association of p-MET with PTP1B in Hs578T cells with or without HGF treatment (Fig. 4C). The above results indicated that association between PTP1B and p-MET was suppressed by THEMIS2. To confirm the overexpression and knockdown of THEMIS2, one tenth of the total lysates were used as input controls and subjected to immunoblotting (Supplementary Fig. 12). Duolink PLA analysis confirmed that depletion of THEMIS2 enhanced the formation of both p-MET-PTP1B and MET-PTP1B complexes in Hs578T and MDA-MB-231 cells with or without HGF treatment (Fig. 4D, Supplementary Fig. 13). The interaction was manifested by increased red fluorescent dots reflecting close proximity interaction between PTP1B and p-MET as well as between PTP1B and MET, as shown by increased PLA puncta for p-MET-PTP1B or MET-PTP1B (Fig. 4E). We also noticed that the strengthening effects on both p-MET-PTP1B and MET-PTP1B interactions were less prominent after stimulation with 30 ng/mL HGF for 30 min compared with the non HGF treated controls (Fig. 4D, Supplementary Fig. 13), which could be due to internalization of the HGF-MET complexes. Taken together, these results suggest that THEMIS2 interferes with the association of PTP1B with p-MET and MET, thereby protecting p-MET from dephosphorylation by PTP1B.

### THEMIS2–P-MET axis was blocked by cryptotanshinone (CPT) in TNBC

**Fig. 5:**
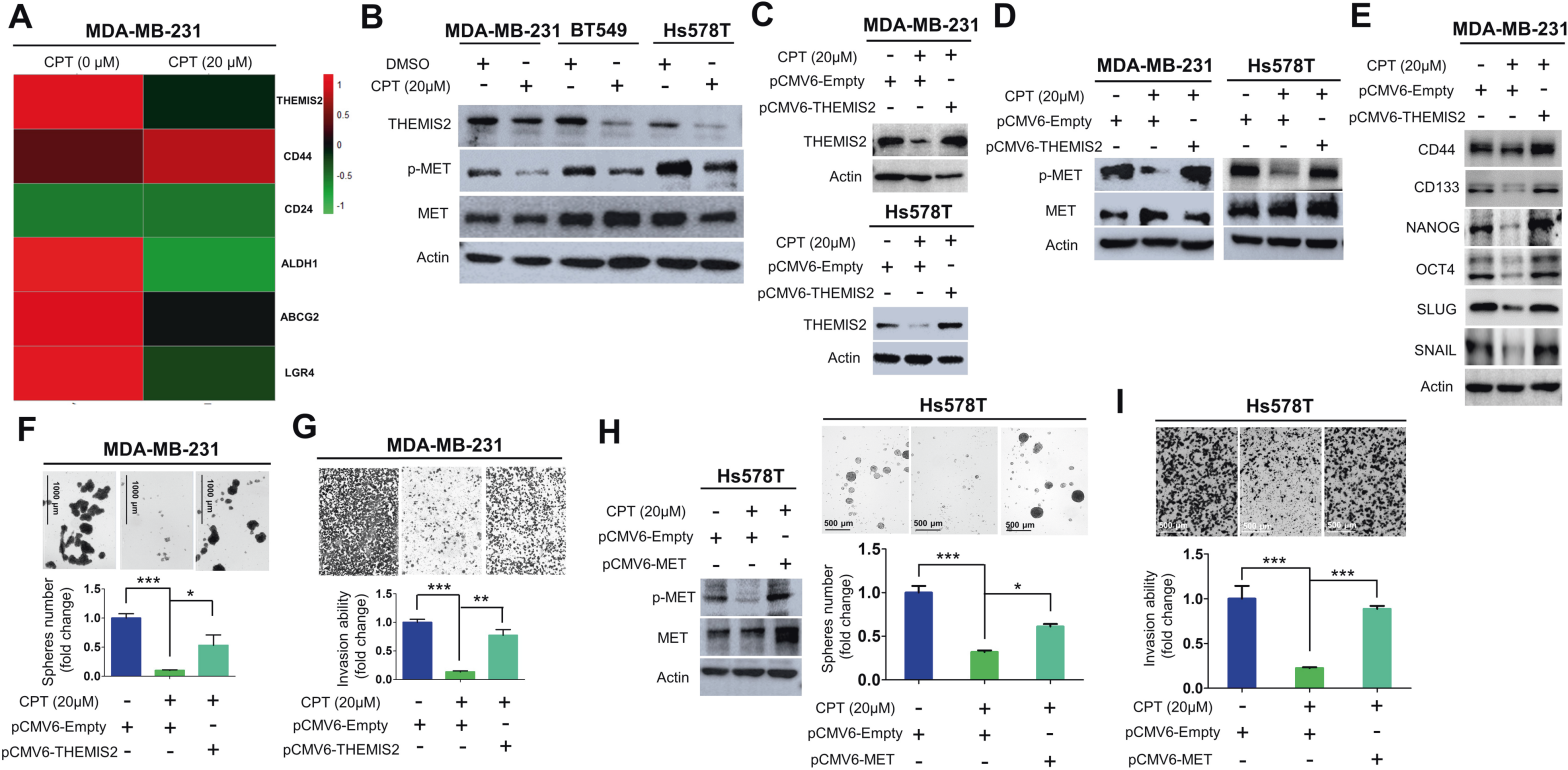
CPT inhibited THEMIS2 expression and THEMIS2-induced cancer stemness, which could be partially rescued by MET. **A** Changes in gene expression of THEMIS2 and CSC markers (CD44, CD24, ALDH1, ABCG2, and LGR4) in MDA-MB-231 cells treated with or without 20 μM CPT are shown in a heat map. The colors red, black, and green are shown on a scale reflecting high to low expression. **B** Protein expression levels of THEMIS2, p-MET, and MET in MDA-MB-231, BT549, and Hs578Tcells 24 h after treatment with CPT (20 μM) as analyzed by immunoblotting. Actin was used as the internal control. **C** Protein expression levels of THEMIS2 in MDA-MB-231 and Hs578T cells transfected with the indicated plasmids as analyzed by immunoblotting. Actin was used as the internal control. **D** Protein expression levels of p-MET and MET in MDA-MB-231 cells transfected with the indicated plasmids were analyzed. Actin was used as the internal control. **E** Protein expression levels of CSC markers (CD44, CD133, Nanog, and OCT4) and EMT-related markers (SLUG and SNAIL) in MDA-MB-231 cells transfected with the indicated plasmids were analyzed. Actin was used as the internal control. **F** The sphere-forming abilities of MDA-MB-231 cells was inhibited after treatment with 20 μM CPT. This inhibition was partially rescued upon the ectopic expression of THEMIS2. **G** The invasion abilities of MDA-MB-231 cells was inhibited after treatment with 20 μM CPT and was rescued through the ectopic expression of THEMIS2. **H** The sphere-forming abilities of Hs578T cells transfected with the indicated plasmids were inhibited after treatment with 20 μM CPT and was partially rescued upon the ectopic expression of MET. **I** The invasion abilities of Hs578T cells transfected with the indicated plasmids was inhibited after treatment with 20 μM CPT. This inhibition was substantially rescued through the ectopic expression of MET. The histograms represent means ± SEs from three independent experiments (*P < 0.05; **P < 0.01; ***P < 0.001). Representative pictures of those functional assays are presented.

A previous report indicated that CPT inhibits angiogenesis by blocking VEGFR2 activation and its downstream signaling pathways [30]. Another study has reported that the downstream signaling molecules of MET receptor including PI3K/AKT, MAPK and STAT3 were regulated by VEGF expression [14]. Moreover, CPT has been reported in a study as being able to inhibit the activity of prostate tumor-initiating cells [31]. On the basis of those findings, we investigated whether CPT could inhibit CSC properties by blocking THEMIS2–p-MET axis and its downstream pathways. We first examined the anti-proliferative effect of CPT on TNBC cells. Cell proliferation was significantly inhibited by CPT at 40 μM, but not at 1 or 20 μM (Supplementary Fig. 14). To test whether CPT could affect THEMIS2 expression and cancer stemness, we treated MDA-MB-231 cells with a relatively low and noncytotoxic dose of CPT (20 μM) and compared the resulting gene expression profile with that of the untreated control cells through RNA-sequencing and GO enrichment analysis. The gene expression of THEMIS2 and several CSC markers including ALDH1, ABCG2 and LGR4 were found downregulated (Fig. 5A) [30, 32]. Downregulation of THEMIS2, ALDH1, ABCG2, and LGR4 and upregulation of CD24 were confirmed by qRT-PCR (Supplementary Fig. 15). Treatment with 20 μM CPT suppressed the protein expression levels of THEMIS2 and the phosphorylation of MET in three TNBC lines (Fig. 5B). Ectopic expression of THEMIS2 reversed the CPT-mediated inhibition of THEMIS2 in stable THEMIS2-expressing MDA-MB-231 and Hs578T cells as shown by immunoblotting analysis (Fig. 5C). The phosphorylation but not the protein level of MET was reduced when MDA-MB-231 and Hs578T cells were treated with 20 μM CPT, whereas ectopic expression of THEMIS2 rescued the CPT-mediated inhibition of MET-activation phosphorylation (Fig. 5D). Treatment with CPT (10 μM) significantly reduced Tyr705 phosphorylation of STAT3 in MDA-MB-231 and Hs578T cells (Supplementary Fig. 16). A previous study indicated that STAT3 increased MET expression via activation of the positive feedback loop [33]. Our data also revealed depletion of THEMIS2 led to reduction of Tyr705-P-STAT3 (Supplementary Fig. 4D). Based on those findings, the effect of CPT is likely to be mediated by inhibition of both THEMIS2 and STAT3. Moreover, CPT treatment of MDA-MB-231 cells led to substantial reductions in various CSC- and EMT-related markers [20, 21, 34], which were completely rescued by the ectopic expression of THEMIS2 (Fig. 5E). Similarly, the sphere-forming and invasion abilities of MDA-MB-231 and Hs578Tcells were strongly inhibited upon treatment with 20 μM CPT, and ectopic expression of THEMIS2 significantly rescued this inhibition (Fig. 5F and G). Furthermore, ectopic expression of MET rescued the CPT-mediated inhibition of sphere formation and cell invasion (Fig. 5H and I). We also performed experiments to assess whether overexpression of TPR-MET (constitutively active MET) could rescue THEMIS2 depletion-mediated effects on TNBC cells. Our data showed that there was a significant reduction of cell invasion and sphere forming ability in the THEMIS2 depleted MDA-MB-231- and Hs578T cells, and transfection with TPR-MET rescued the THEMIS2 depletion-mediated suppression of cell invasion and sphere formation by approximately 60% and 70%, respectively (Supplementary Fig. 17). These results supported that MET is able to rescue most of the THEMIS2-depeleted-mediated inhibition. In short, in TNBC, CPT potently inhibited cancer stemness properties and cell invasion through the downregulation of the THEMIS2/MET signaling pathway.

### Overexpression of THEMIS2 sensitizes TNBC for MET targeting drug

**Fig. 6:**
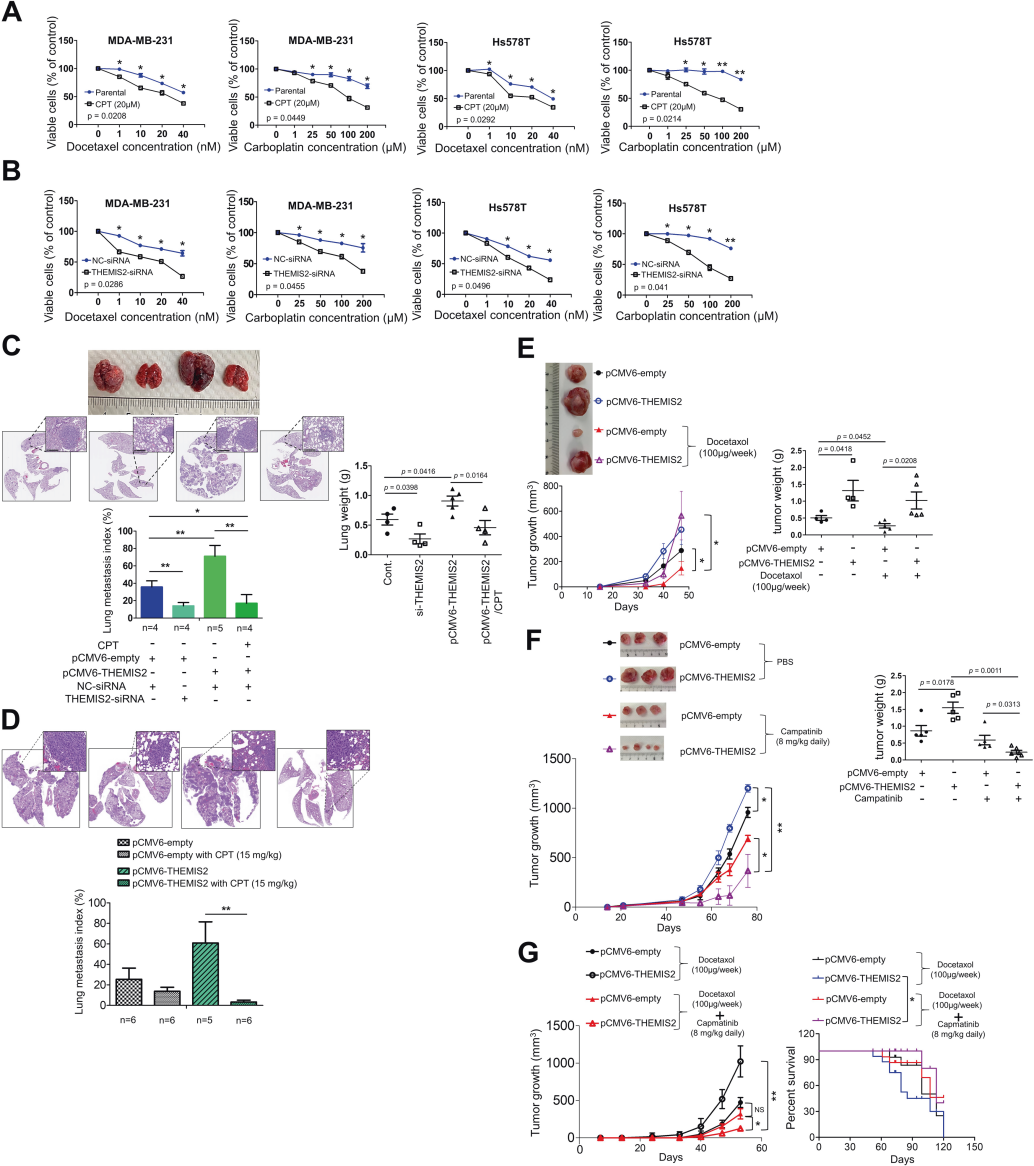
Effect of CPT, Capmatinib and THEMIS2 on the chemosensitivity, pulmonary metastasis and tumor growth in TNBC cells. **A** Dose-dependent growth inhibition of MDA-MB-231 and Hs578T cells under continuous exposure to the indicated concentrations of docetaxel or carboplatin for 24 h was measured using an MTT assay. Cells were divided into two groups according to treatment with CPT (20 μM) or dimethyl sulfoxide. **B** Dose-dependent growth inhibition of MDA-MB-231 and Hs578T cells transfected with control or THEMIS2 siRNA for 8 h, serum starved for 24 h, and upon continuous exposure to the indicated concentrations of docetaxel or carboplatin for 24 h was measured using an MTT assay. **C** The lung weights of four groups of CB17-SCID mice that had received tail vein injections of MDA-MB-231-IV2 cells that had been transfected with the indicated siRNAs or plasmids with or without treatment with CPT (15 mg/kg) were compared using a two-tailed Mann–Whitney test. The scatter plot represents means ± SEs from five mice in each group (*P < 0.05; **P < 0.01). Representative pictures of the pulmonary metastatic tumors and lung metastasis index of the mentioned four mice groups are shown; specimens were stained with hematoxylin and eosin. **D** Lung metastasis of mice, which were tail vein injected with 1 × 10^6^ MDA-MB-231 cells followed by treatment with or without CPT (15 mg/kg). **E** Docetaxel treatment substantially reduced tumor size, whereas THEMIS2 overexpression increased chemoresistance in vivo (*n* = 5 for each group). Left: tumor growth was monitored; right: CB17-SCID mice were categorized into four groups according to the treatment with docetaxel (100 μg/week) as indicated. In each group, 5 × 10^5^ Hs578T-FPI cells were injected into the mammary fat pads of CB17-SCID mice. **F** Capmatinib treatment substantially reduced tumor size, whereas THEMIS2 overexpression further increased the drug sensitively. Tumor growth was monitored. CB17-SCID mice were categorized into four groups according to the treatment with Capmatinib (8 mg/kg daily) with or without THEMIS2 over expression as indicated. In each group, 1 × 10^6^ MDA-MB-231 cells stably transfected with the indicated plasmids were injected into the mammary fat pads of CB17-SCID mice (*n* = 6 for each group). The quantification of tumor weight of the excised tumors of all treatment groups was performed. **G** Left, CB17-SCID mice were orthotopically injected with 1×10^6^ MDA-MB-231 cells followed by treatment with docetaxel alone or in combination with Capmatinib. Tumor growth was monitored in four groups as indicated (*n* = 10 for each group). Right, survival analysis of the four indicated groups. All mice were sacrificed 30-60 days after implantation. All in vitro experiments (A and B) were performed in triplicate and repeated three times (*P < 0.05). The data in **C**, **D**, **E**, **F**, **G** represent means ± SEs from two to three independent experiments (*P < 0.05; **P < 0.01).

The chemotherapeutic agents for TNBC frequently used today include taxanes and platinum compounds that target the DNA repair complex [3]. Thus, we investigated the effect of CPT and THEMIS2 on the chemosensitivity of TNBC cells toward taxanes and platinum drugs. MDA-MB-231 and Hs578T cells were treated with docetaxel or carboplatin with or without addition of 20 μM CPT. A MTT assay revealed that the TNBC cells became more chemosensitive to continuous exposure to various doses of these drugs under combined treatment with CPT; such a combination treatment exerted an enhanced anti-tumor activity (Fig. 6A). Considering that CPT inhibited cancer stemness by suppressing THEMIS2 expression and MET phosphorylation activation, we investigated whether the inhibition of endogenous THEMIS2 expression could affect the chemosensitivity of TNBC cells with effects mimicking those of the CPT treatment. As expected, silencing THEMIS2 by siRNA significantly increased chemosensitivity toward docetaxel or carboplatin in MDA-MB-231 and Hs578T cells (Fig. 6B).

Next, we tested the effect of CPT on THEMIS2 promoted TNBC cancer metastasis. We employed an experimental metastasis model in which 1 × 10^6^ MDA-MB-231-IV2 cells with THEMIS2 overexpression or knockdown were injected into the tail veins of CB17-SCID mice. In one of the two THEMIS2 overexpression groups, CPT (15 mg/kg) was administered by intraperitoneal injection for 7 weeks, starting 1 week after the tumor cells injection. Knockdown or ectopic expression of THEMIS2 resulted in reduced or increased pulmonary metastasis, respectively, and CPT treatment greatly mitigated the THEMIS2-augmented lung metastasis (Fig. 6C). We then analyzed the expression levels of THEMIS2 and ALDH1 in each of the four treatment groups tumors through IHC staining. THEMIS2 overexpression induced the elevated expression of ALDH1, and treatment with CPT significantly reduced expression of both THEMIS2 and ALDH1 (Supplementary Fig. 18). These results indicate that treatment with CPT reduces lung metastasis through suppressing THEMIS2 expression and cancer stemenss. Interestingly, elevated expression of THEMIS2 rendered tumors more sensitive to CPT treatment (Fig. 6D), suggesting that CPT could be used to inhibit lung metastasis through downregulation of THEMIS2 and MET signaling.

To further explore the role of THEMIS2 in chemoresistance in vivo, we employed an experimental orthotopic model in which Hs578T-FPI cells were injected into the mammary fat pads of CB17-SCID mice. As expected, overexpression of THEMIS2 significantly enhanced tumor growth. Treatment with docetaxel (100 μg/week) significantly inhibited tumor growth and reduced tumor weight (Fig. 6E). However, overexpression of THEMIS2 effectively overcame the inhibition of docetaxel treatment, suggesting that THEMIS2 plays a crucial role in chemoresistance (Fig. 6E). Notably, we observed increased THEMIS2 and p-MET levels in docetaxel and carboplatin treated TNBC cells (Supplementary Fig. 19). To define the effect of THEMIS2 on the response to MET therapeutic, we choose Capmatinib, a highly selective MET inhibitor which blocks ATP binding, for the treatment. Our study showed that increased p-MET protein expression in stably THEMIS2-overexpressing MDA-MB-231 and Hs578T cells (Supplementary Fig. 20A, left). Increased chemosensitivity towards Capmatinib was observed in stably THEMIS2-overexpressing MDA-MB-231 and Hs578T cells when compared with the control cells (Supplementary Fig. 20A, right). Consistently, mice injected with stably THEMIS2-overexpressing MDA-MB-231 cells displayed faster tumor growth than those injected with the control MDA-MB-231 cells, however, the former were more sensitive to Capmatinib treatment than the control group (Fig. 6F). Similarly, mice injected with stably THEMIS2-overexpressing MDA-MB-231 cells displayed larger tumor growth than those injected with the control cells upon treatment with docetaxel, yet the former were more sensitive to the combination therapy with docetaxel and Capmatinib than the control group (Fig. 6G). These results imply that THEMIS2-mediated upregulation of MET signaling sensitizes the tumors towards MET targeted therapy mimicking the “oncogene addiction” and “oncogenic shock” phenomenon [35, 36]. This is consistent with the recent studies indicating that MET inhibitor could be used more effectively for cancer patients with high MET expression or MET amplification [37–39]. Thus high expression of THEMIS2 could be a potential indicator for effective MET targeting therapy in TNBC patients. In addition, we assessed the MET inhibitors sensitivity of Hs578T cells transiently transfected with THEMIS2. The THEMIS2 transiently transfected TNBC cells indeed did not display increased sensitivity toward the 3 MET inhibitors (Supplementary Fig. 20B*)*. This further supports the notion that stable THEMIS2 overexpression cells are more sensitive to the MET inhibitor resulting from THEMIS2 induced prolonged MET activation and its oncogenic addiction.

### Correlations between THEMIS2 expression levels and clinical outcomes in patients with breast cancer and positive correlation between THEMIS2 and p-MET

**Fig. 7:**
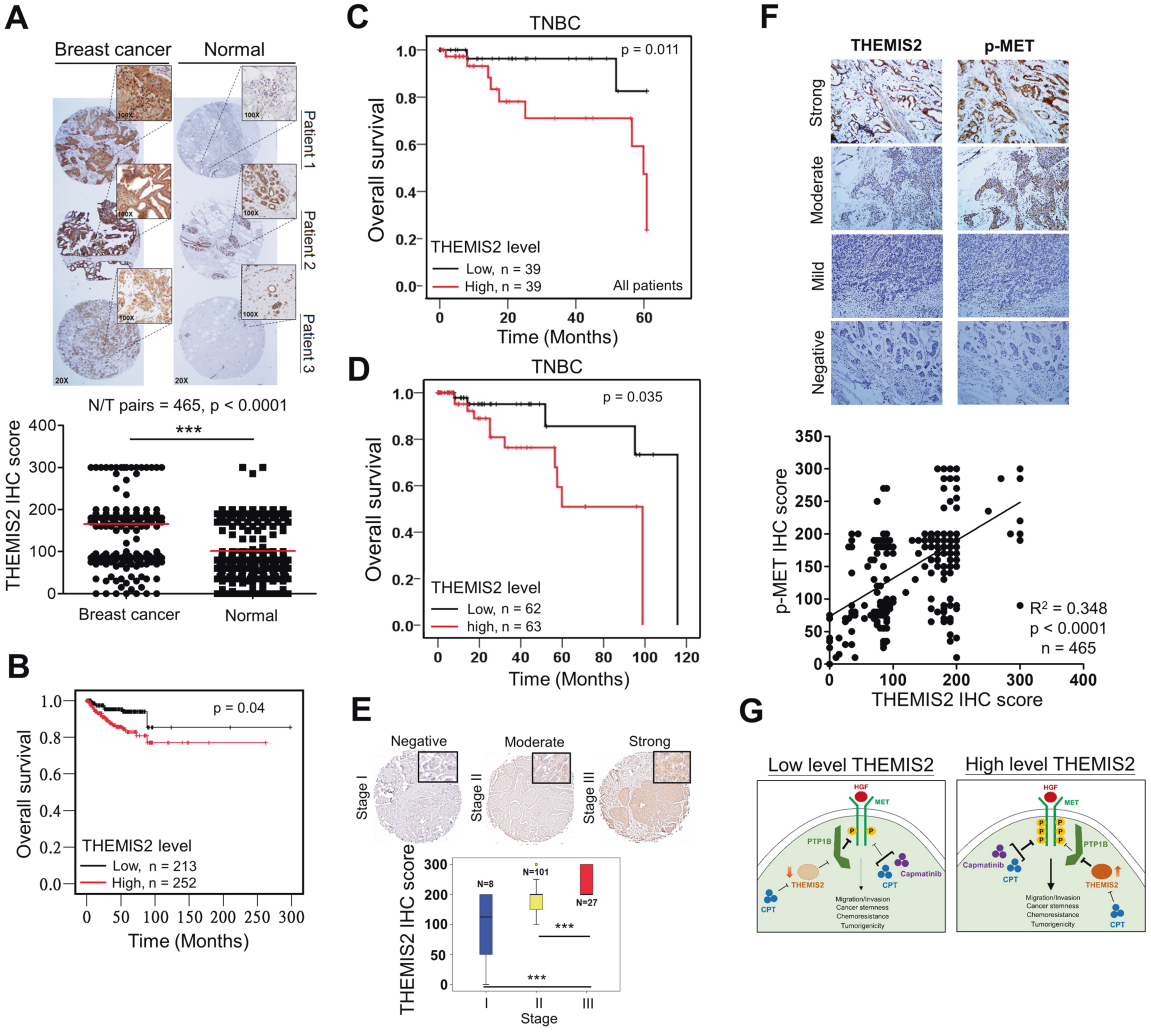
Association of THEMIS2 expression levels with clinical outcomes in patients with breast cancer. **A** THEMIS2 protein levels in 465 pairs of breast cancer tissues and their adjacent normal tissues were examined using IHC staining (***P < 0.001). **B** THEMIS2 protein levels were correlated with overall survival in all 465 patients. Kaplan–Meier analysis of Oncolink (https://www.oncolink.org/cancers/breast) (**C**) and Oncomine database (TCGA Breast and TCGA Breast 2, https://www.oncomine.org/resource/login.html) (**D**) for TNBC revealed that patients with high expression of THEMIS2 had worse overall survival compared to the THEMIS2 low expression patients. **E** The THEMIS2 protein levels were determined using IHC staining in a commercial tissue array slide containing 136 TNBC specimens. Representative images of tissue from patients with stages I, II, and III cancer are presented. The floating column chart represents means ± SEs from three independent experiments (***P < 0.001). **F** THEMIS2 and p-MET protein levels in the 465 specimens were positively correlated. **G** The proposed model of high level THEMIS2 may sensitize CPT and Capmatinib in TNBC. In brief, THEMIS2 enhances cancer stemness, chemoresistance and tumorigenicity by suppressing p-MET–PTP1B interaction to activate MET phosphorylation.

Finally, we explored the correlations between THEMIS2 expression levels and clinical outcomes of breast cancer patients. The THEMIS2 protein levels in 465 pairs of breast cancer tissue specimens and the adjacent normal tissues from our local cohort were determined through IHC staining. The IHC score of THEMIS2 was significantly higher in the breast cancer tissues than in the normal tissues (Fig. 7A). Kaplan–Meier survival analysis of the 465 cases revealed that breast cancer samples with higher THEMIS2 levels were correlated with significantly poorer survival than were those with lower THEMIS2 levels (Fig. 7B). Moreover, the correlation between THEMIS2 mRNA expression and the overall survival of breast cancer patients was assessed through a web server program, as described in the Methods section. Our data assessing the correlation between THEMIS2 mRNA expression (used the auto select best cutoff) and the overall survival of breast cancer patients (210785_s_at) by Kaplan-Meier Plotter revealed no significant correlation (*p* = 0.35, Supplementary Fig. 21, *left*) in the earlier version of data base (https://kmplot.com/analysis/, 01 January 2020). Moreover, high THEMIS2 expression correlated with shorter overall survival in patients (210785_s_at) who received chemotherapy (excluding the patients of endocrine therapy) with regard to their drug treatment modalities (*p* = 0.025, Supplementary Fig. 21, *right*) in the earlier version of data base (https://kmplot.com/analysis/, 01 January 2020). Now the *p* values of overall survival of all breast cancer patients (210785_s_at) and those received chemotherapy (excluding those with endocrine therapy) in statistics have been updated to 0.29 and 0.11, respectively, in the updated data base (https://kmplot.com/analysis/, 01 August 2021) (Supplementary Fig. 21, *bottom*). Although the survival benefit with regard to THEMIS2 for breast cancer patients who received chemotherapy diminished in the updated data base in the KM Plotter analysis, we did find that overexpression of THEMIS2 correlated with poor prognosis in TNBC patients by Oncolink database (https://www.oncolink.org/cancers/breast, Fig. 7C) and Oncomine database (https://www.oncomine.org/resource/login.html, Fig. 7D). One previous report indicated that higher expression of THEMIS2 correlated with better relapse-free survival as well as overall survival in basal-like subtype of breast cancer patients which resemble TNBC patients [40], the inconsistent analytic results might be due to the use of different database and the heterogeneous characteristics of TNBC. We also examined THEMIS2 expression in a commercial tissue array slides containing 136 TNBC specimens through IHC staining. The THEMIS2 expression levels were higher in patients with stage III TNBC than in those with stage I/II TNBC (Fig. 7E).

Because no studies have reported correlations between THEMIS2 and MET activity in breast cancer thus far, we assessed their clinical connection by examining THEMIS2 and p-MET protein levels in breast cancer tissue specimens. Levels of THEMIS2 and p-MET protein were significantly correlated in the 465 breast cancer specimens (Fig. 7F). These collective results support the premise that THEMIS2 contributes crucially to the regulation of chemoresistance, and that it is correlated with poor clinical outcomes in TNBC patients.

## Discussion

In this study we have identified THEMIS2 as a novel modulator of cancer stemness and chemoresistance, revealed its regulatory mechanism in MET activation, and suggested a relevant clinical application using CPT or Capmatinib to target THEMIS2/MET axis. Cancer stem cells are known to play important roles in cancer initiation, metastasis, recurrence and chemoresistance [32, 41]. TNBC was chosen for our endeavor to explore CSC regulatory genes and potential therapeutic targets due to poor prognosis and lack of targeted therapy for this type of cancer. THEMIS2 has been reported to be involved in estrogen responses in ovarian and breast cancer cells [5, 7]. Although a previous study indicated that THEMIS2 might act as a tumor suppressor and reduced THEMIS2 level exhibited relative resistance to apoptotic drugs such as tamoxifen and staurosporine in MCF-7 breast cancer cells [42], our data revealed the opposite results to demonstrate that THEMIS2 exerted the oncogenic function to promote cell proliferation and sphere formation in TNBC cells (Supplementary Fig. 3). The inconsistent findings between our data and those of Treeck’s study may be due to differences in hormone receptor status in breast cancer cells. Since MCF-7 cells possess both estrogen and progesterone receptors and our data are mainly applied to TNBC cells, lack of hormone receptors might affect the regulatory function of THEMIS2 in breast cancer cells and warrants further investigation. To test that THEMIS2 might regulate hormone receptor positive breast cancer cells, such as MCF7, we performed an additional MTT assay and observed knockdown or overexpress THEMIS2, increase or decrease slightly, respectively, the growth of MCF-7 cells (Supplementary Fig. 22). The results are consistent with those of Treeck et al.’s study and also affirmed that THEMIS2 appeared to regulate breast cancer cells differently with respect to status of hormone receptors. The underlying mechanism is interesting and warrants further investigation. In addition, THEMIS2 could be a potential biomarker and target for breast cancer treatment guidance with respect to ER status.

Nevertheless, prior to this study, THEMIS2 has seldom been discussed with respect to TNBC and had not been implicated in MET inhibitor resistance. Our study indicates that THEMIS2 is a potential novel CSCs therapeutic target via activation of MET signaling. Capmatinib, as a highly potent and selective inhibitor of MET, has been shown to be effective as a single-agent treatment in patients with high-level MET-amplified advanced NSCLC or in patients with advanced or metastatic NSCLC harboring *MET*ex14 [16, 43]. Nevertheless, no evidence has shown that Capmatinib can be applied in the treatment of TNBC yet. In our present work, we found that overexpression of THEMIS2 increases the synergistic effects of docetaxel and Capmatinib in vivo (Figs. 6E, 6F and 6G, and Supplementary Fig. 19). Capmatinib may worth further study in treating TNBC patients with overexpression of THEMIS2 through targeting THEMIS2/MET signaling.

Although the high level of MET protein is associated with poor prognosis in cancer, it was less effective as a predictive biomarker for targeted therapeutic efficacy. Previous studies have found that MET overexpression has higher sensitivity toward MET inhibitors [38, 39], however, due to the significant intratumoral heterogeneity, immunohistochemical evaluation of MET overexpression remains challenging, limiting its usage as a biomarker in the clinic. This difficulty in screening patients for MET-targeted therapy may also partly explain the failure of MET inhibitors in recent phase III clinical trials of NSCLC. It is worthwhile to note that THEMIS2 enhanced MET phosphorylation by perturbing association of PTP1B with p-MET in TNBC (Fig. 4). Because Capmatinib therapy showed efficacy in patients with NSCLC and MET is well established in the etiology of this cancer type, it would be of interest to further determine whether the THEMIS2-MET axis also exists in this tumor type. Our data revealed that elevated THEMIS2 expression in TNBC cells led to MET activation and sensitized them toward CPT and MET inhibitor Capmatinib. Our study suggests that Capmatinib and CPT can be recommended as a combination therapy during and/or subsequent to initial conventional treatment of TNBC. PTP1B has been reported to negatively regulate MET by binding to twin tyrosine (Tyr-1234/1235)-phosphorylated MET in the activation loop of the kinase domain, leading to its dephosphorylation and inactivation [28, 44]. In one study, PTP1B regulated the endosomal trafficking of MET after HGF stimulation [44]. THEMIS2 depletion also significantly increased the binding of PTP1B to p-MET, whereas THEMIS2 overexpression had the opposite effect. Because THEMIS2 enhanced the phosphorylation of MET but had no effect on the MET protein level (Fig. 3), and because THEMIS2 appeared to interfere with the association of PTP1B and p-MET, we postulate that THEMIS2 may compete with PTP1B for binding to p-MET, thereby protecting it from dephosphorylation by PTP1B. The specific THEMIS2-mediated enhancement of MET phosphorylation activation, but not of the other RTKs tested, could be due to the specific interaction between THEMIS2 and MET, which could perturb the association of PTP1B with MET. Further research is required to confirm this supposition.

In summary, we have identified THEMIS2 as a novel regulator of cancer stemness and chemoresistance through interfering with the association of PTP1B with p-MET to promote MET signaling in TNBC cells. The potential application of CPT and Capmatinib in the treatment of TNBC via suppression of THEMIS2/MET signaling was also identified in our study providing a new therapeutic direction in the future (Fig. 7G).

## Materials and methods

### Patients

A total of 465 breast cancer tissue microarray slides were obtained from patients undergoing surgery for breast cancer at Show Chwan Memorial Hospital, Taiwan, from July 2011 to November 2019. Pathological examination and further biological analyses were conducted with informed consent. All samples and clinical information were acquired in accordance with study protocol, which was approved by the hospital’s institutional review board (number: 1081102). Immunohistochemistry (IHC) was performed to detect THEMIS2 and p-MET expression in paraffin-embedded breast cancer specimens. The slides were stained with anti-THEMIS2 and anti-p-MET antibodies (Abcam, ab68141, and ab236975), which were purchased from Abcam, Inc. The IHC scores are detailed in the “Immunohistochemistry scoring” section.

### Web server survival analysis

Detailed information of Web server survival analysis in this study is provided in Supplementary Materials and Methods.

### Cell culture and sphere forming assay

Detailed procedure was described elsewhere [13, 45] and Supplementary Materials and Methods. MDA-MB-231-IV2 cells were selected by injection of the MDA-MB-231 cells into tail vein of CB17-SCID mice and isolating lung metastasis to derive the highly metastatic subline and named IV2. Cell lines were found to be mycoplasma free and authenticated by the assessment of short tandem repeat loci profiling.

### Cell proliferation assay

Detailed procedure was described elsewhere [13].

### Transfection of indicated small interfering RNA and the expression vector

Detailed procedure was described in Supplementary Materials and Methods.

### Co-immunoprecipitation assay and immunoblotting analysis

Detailed procedure was described in Supplementary Materials and Methods.

### Cell chemotactic migration and invasion assay

Detailed procedure was described elsewhere [11].

### Soft agar assay

Detailed procedures of soft agar assay were described elsewhere [11].

### Immunofluorescence microscopy

Detailed procedures of immunofluorescence were described elsewhere [11].

### Duolink proximity ligation assay

Detailed procedure was described in Supplementary Materials and Methods.

### Immunohistochemistry (IHC) scoring

Detailed procedure was described in Supplementary Materials and Methods.

### Transcriptome sequencing

Detailed procedure was described in Supplementary Materials and Methods. The clean data were aligned to the reference genome as described previously [46].

### In vivo tumorigenicity and metastasis assays

An in vivo metastasis assay was performed, in which Hs578T-FPI cells (5 × 10^5^) transfected with THEMIS2-overexpressing or control vector were suspended in PBS and injected individually into the tail vein of 6- to 8-week-old female CB17-SCID mice. 1 × 10^6^ MDA-MB-231-pCMV6-THEMIS2-transfected stable cells and 1 × 10^6^ pCMV6-empty-vector-transfected cells were injected individually into the mammary fat pads of 6-to-8-week-old CB17-SCID mice. After implantation of those cells for one week, docetaxel (Merck) and/or Capmatinib (Selleckchem) reagent was intraperitoneally injected 3 times a week until 2 months, respectively. All mice were monitored and sacrificed 30-60 days after implantation. Tumor spread was observed through live animal bioluminescent imaging (Caliper IVIS system, PerkinElmer). Moreover, MDA-MB-231-IV2 cells with or without THEMIS2 overexpression were intravenously injected into the tail veins of 6-to-8-week-old female CB17-SCID mice, which were then monitored for 30–60 days before being sacrificed. Lung tissues were removed, fixed, paraffin embedded, serially sectioned, and subjected to hematoxylin and eosin staining. In the experiments involving CPT treatment, the drug was dissolved in ethylene glycol and further diluted in PBS for weekly *intraperitoneal* (IP) injection with 15 mg/kg CPT or PBS over 7 weeks. The CPT injections were initiated from 14 days to 7 weeks after cell implantation.

In the tumorigenicity assay, 5 × 10^2^ MDA-MB-231-pCMV6-THEMIS2-transfected stable cells and 5 × 10^2^ pCMV6-empty-vector-transfected cells were injected individually into the mammary fat pads of 6-to-8-week-old female CB17-SCID mice randomly. To minimize the sacrifice of the laboratory animals, the sample size of each group was determined to ensure a high likelihood that statistical significance would be observed. Tumor volumes were measured once a week, and tumor growth was evaluated until 4 months after implantation. The experiments were conducted in an exp designer and observer-blinded manner. All procedures were performed in accordance with the Guide for Care and Use of Laboratory Animals issued by the Institutional Animal Care and Use Committee of China Medical University, Taiwan (CMUIACUC-2019-289). All animal experiments were conducted following the protocols approved by the Institutional Review Committee (Approval date: 5 July 2019).

### Statistical analysis

The RNA sequencing data were subjected to EdgeR analysis to determine genes with significant differential expression according to the following criteria: a >2-fold change and a <0.05% false discovery rate between the parental and sphere-derived cells. The GraphPad Prism software (GraphPad Software, CA) was used to generate graphs and two-tailed paired or unpaired t-tests were performed to determine the significance between the groups compared. Except where otherwise noted, data presented in figures are shown as mean ± standard deviation (SD). Independent samples *t* tests were conducted using SPSS Statistics for Windows, version 18.0 (SPSS Inc., Chicago, IL, USA) to determine differences in the frequencies and means of the categorical data, *P* values of <0.05 were considered statistically significant.

## Supplementary information

Supplementary Figures

Supplementary Figure legends

Supplementary Table 1

Supplementary Materials
