# Supplementary material for: Novel function of THEMIS2 in the enhancement of cancer stemness and chemoresistance by releasing PTP1B from MET
Source: Oncogene. 2022 Jan 1;41(7):997–1010. doi: 10.1038/s41388-021-02136-2 (PMC8837547; doi:10.1038/s41388-021-02136-2)

**Supplementary Figure 1**

**
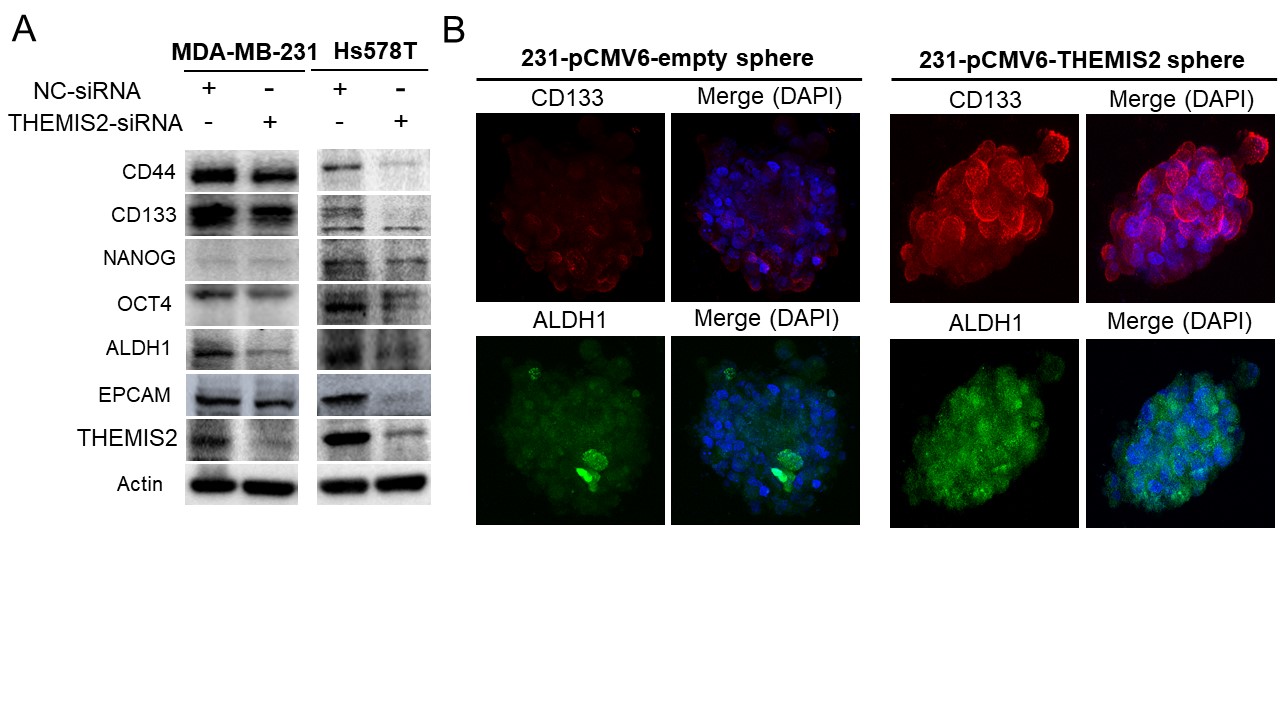
**

**Supplementary Figure. 2**


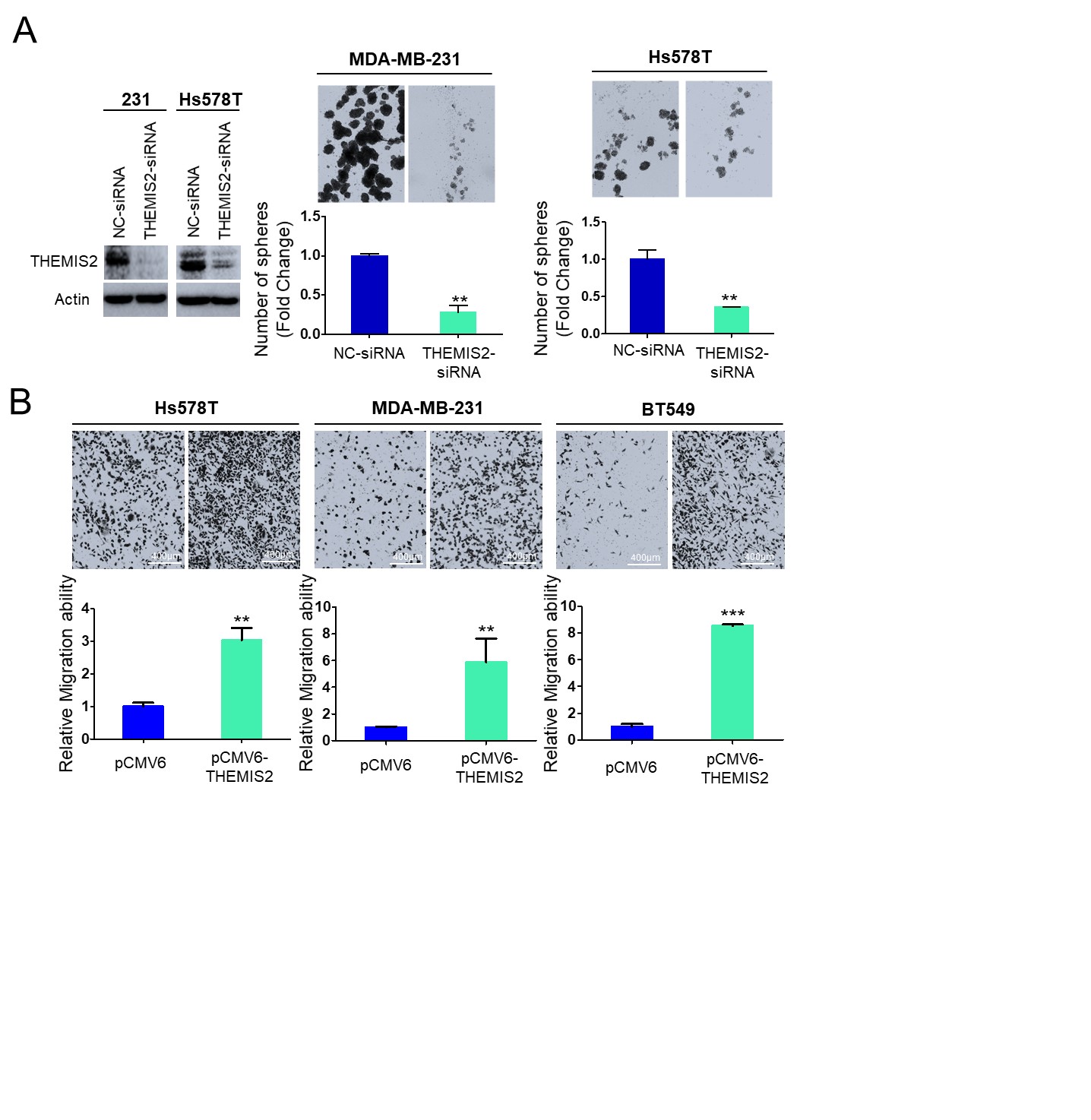


**Supplementary Figure. 3**


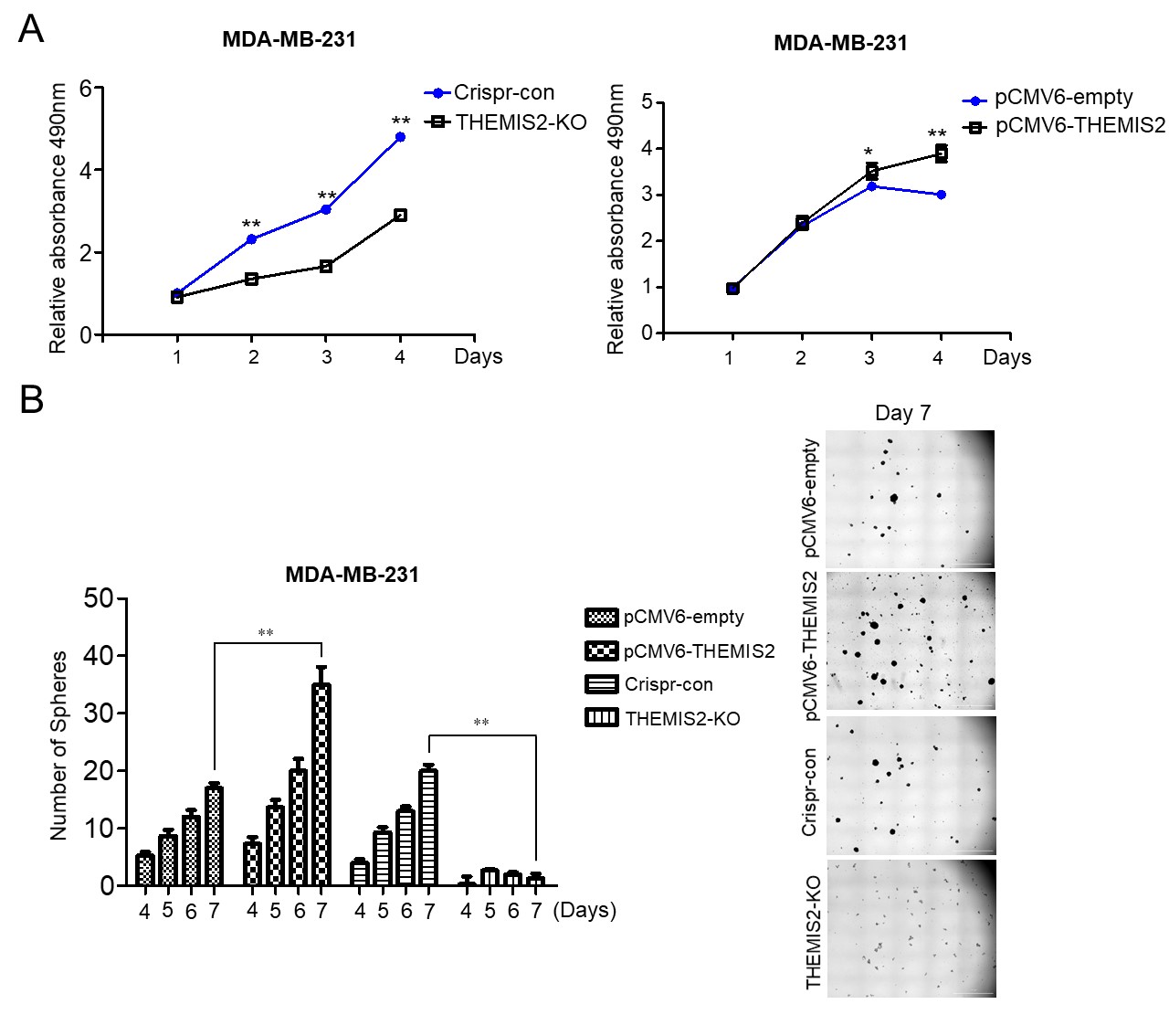


**Supplementary Figure. 4**


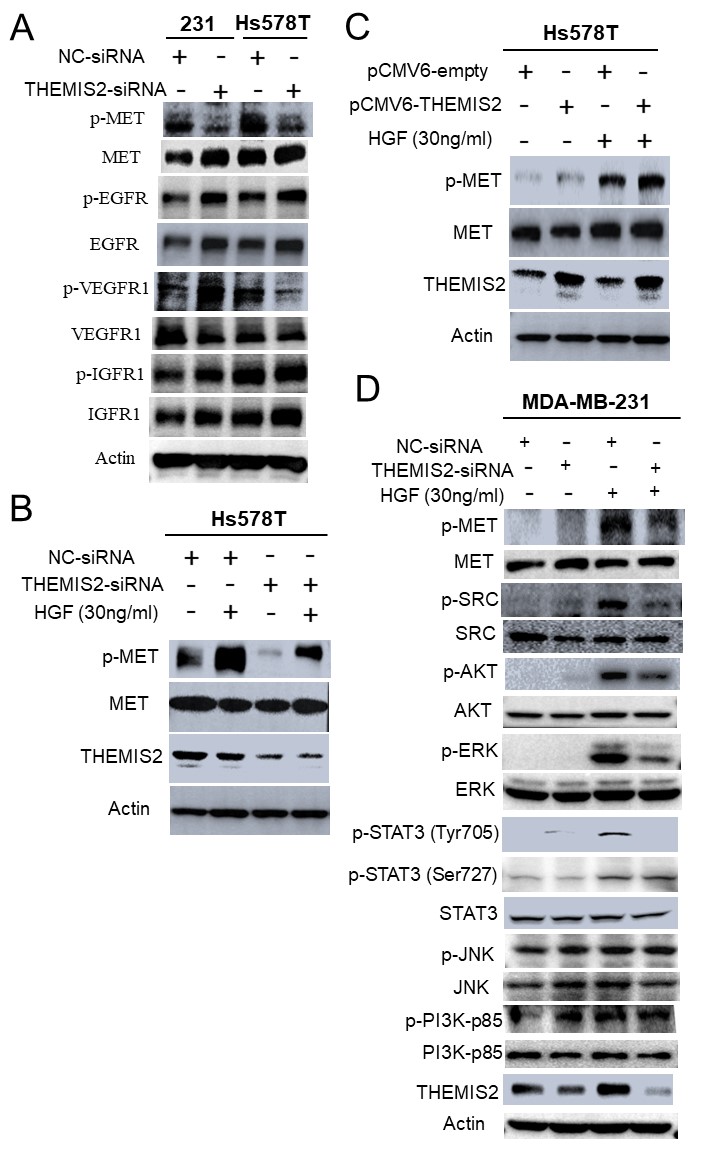


**Supplementary Figure. 5**


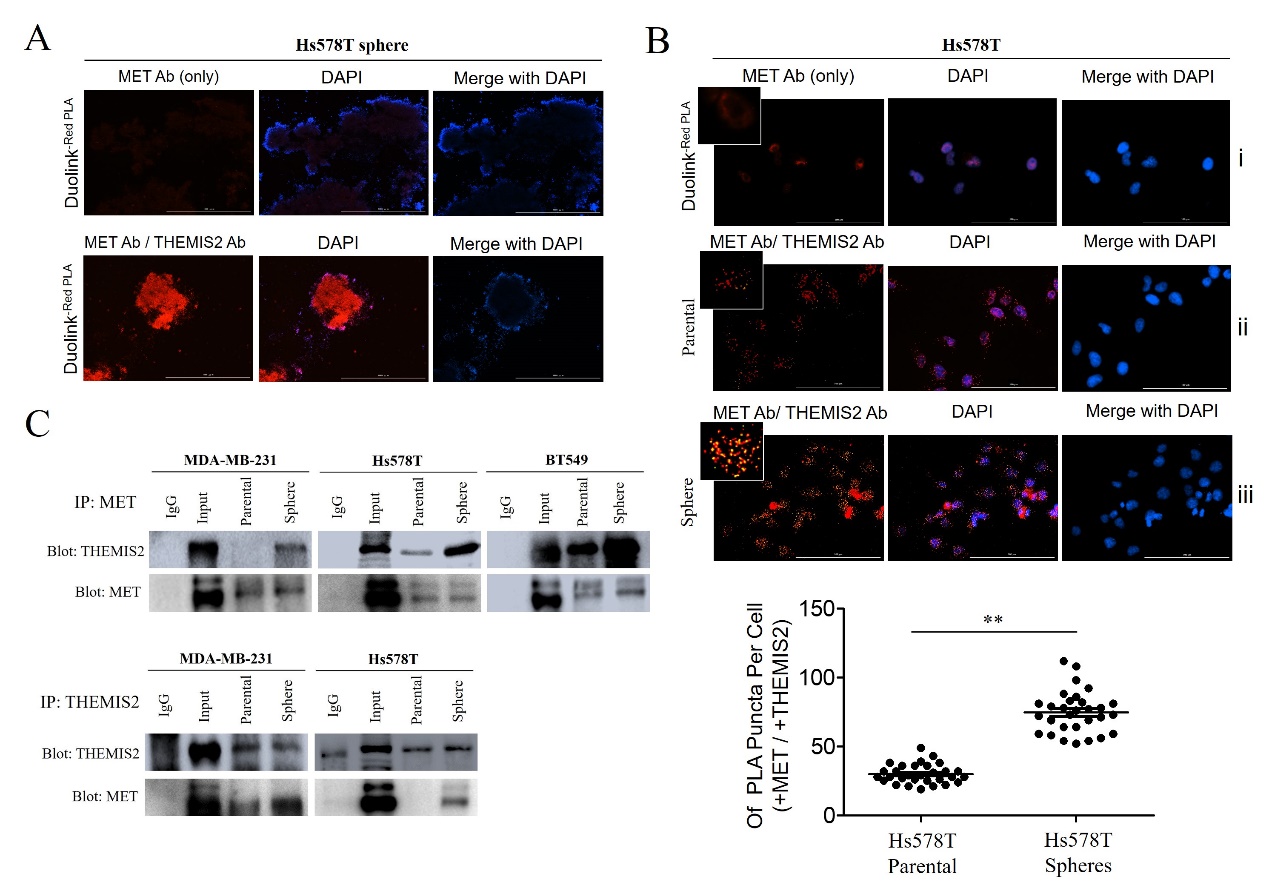


**Supplementary Figure. 6**


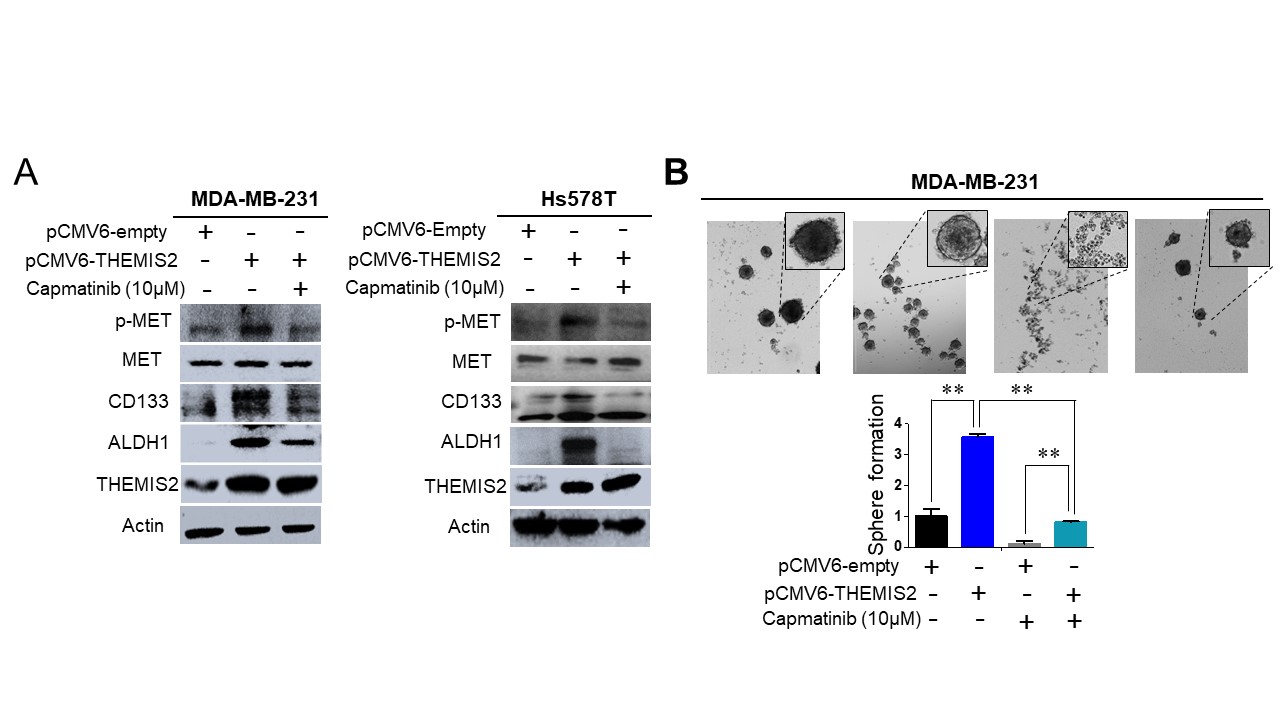


**Supplementary Figure. 7**


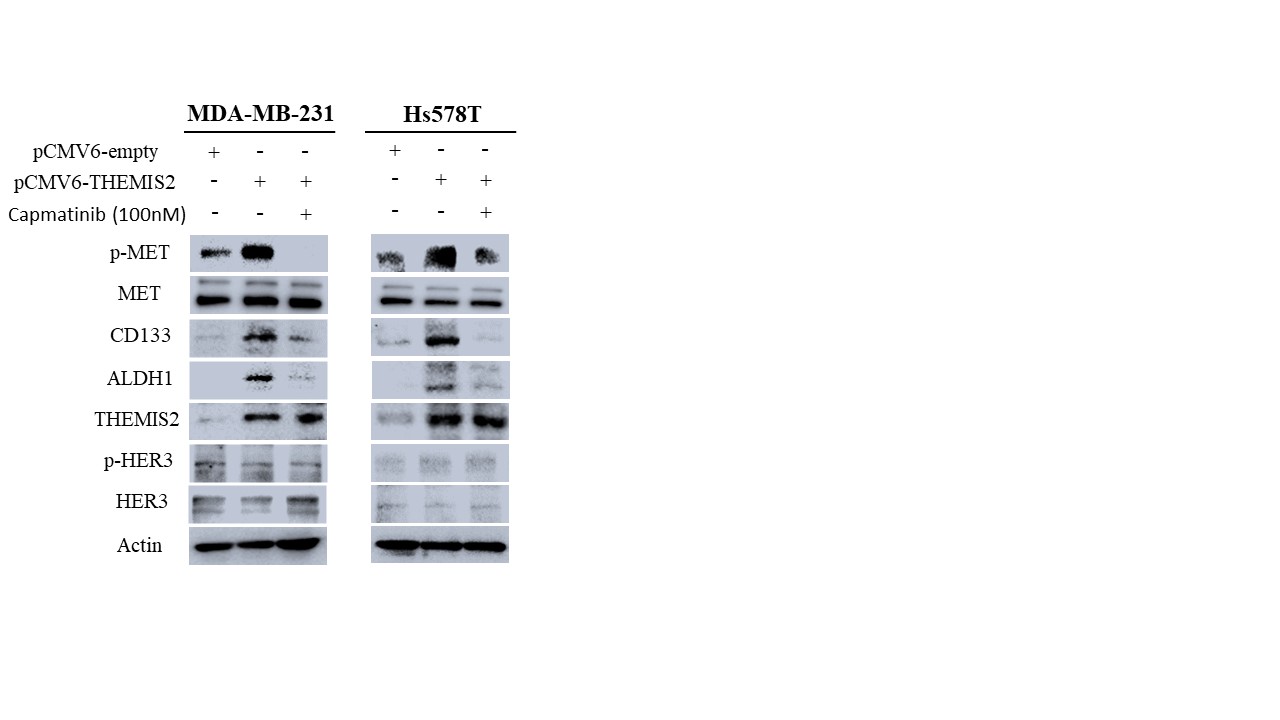


**Supplementary Figure. 8**


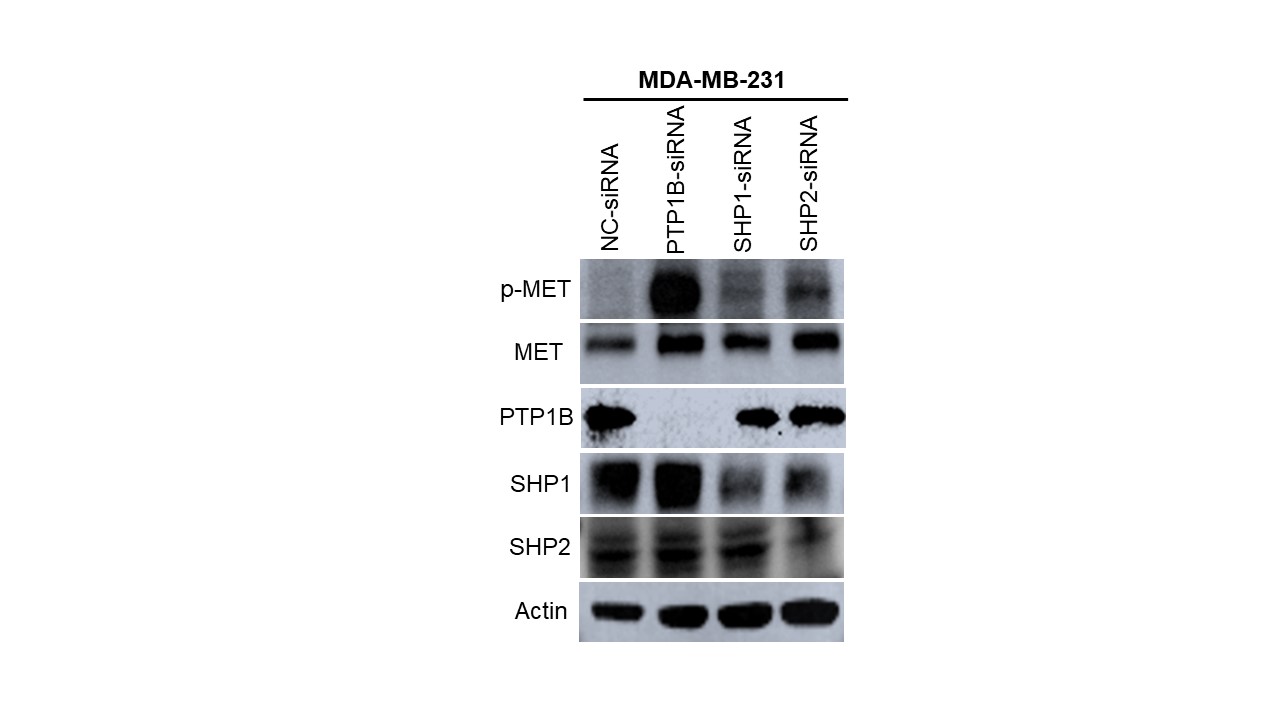


**Supplementary Figure. 9**

**
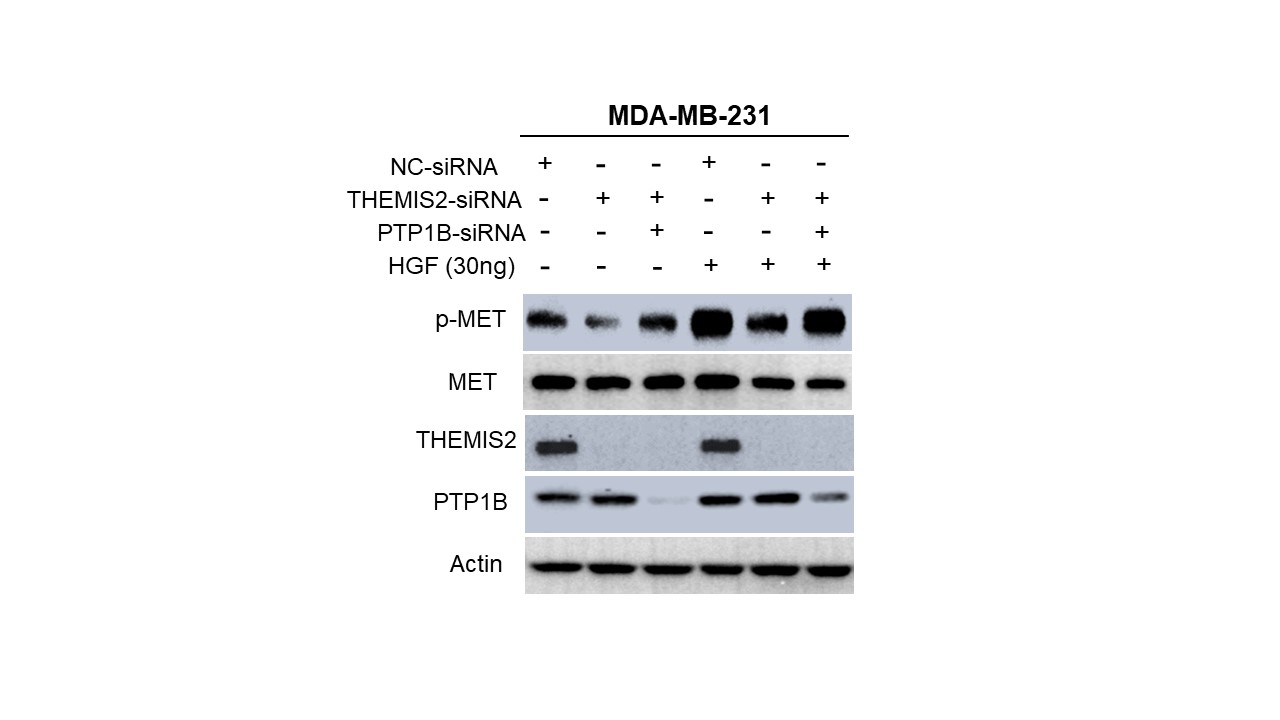
**

**Supplementary Figure. 10**


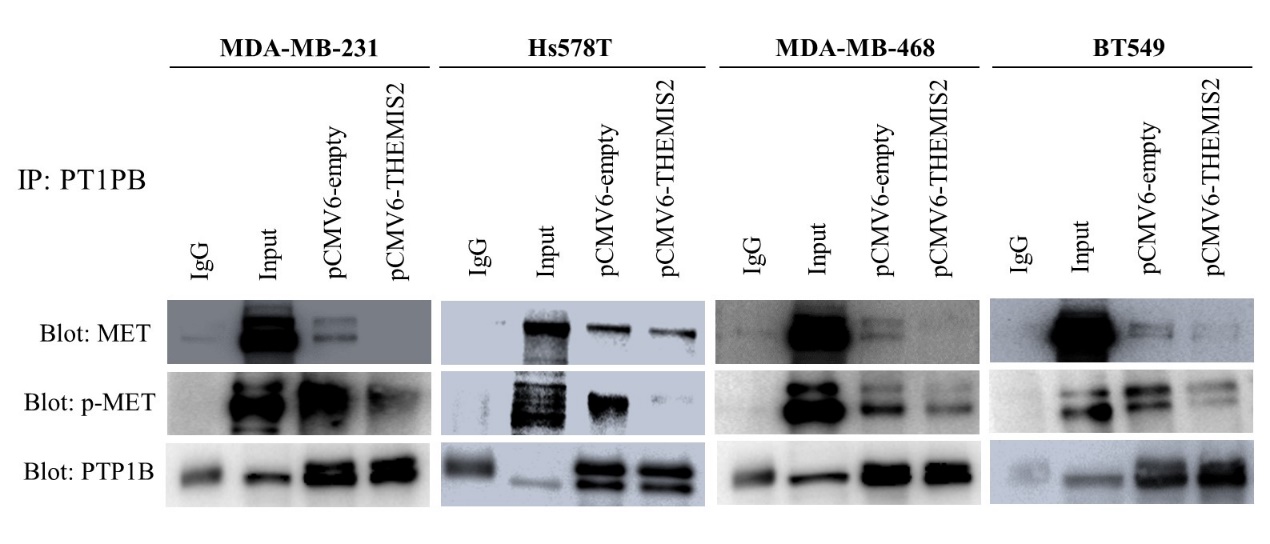


**Supplementary Figure. 11**


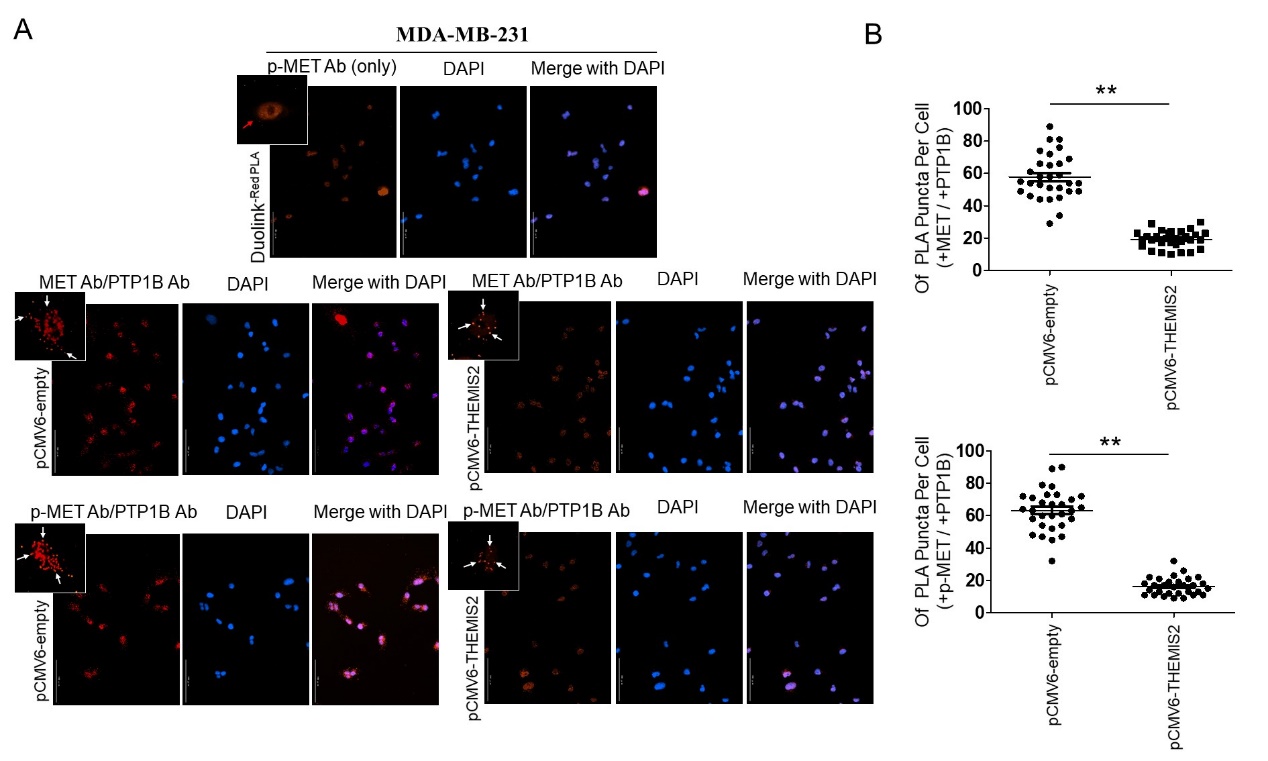


**Supplementary Figure. 12**


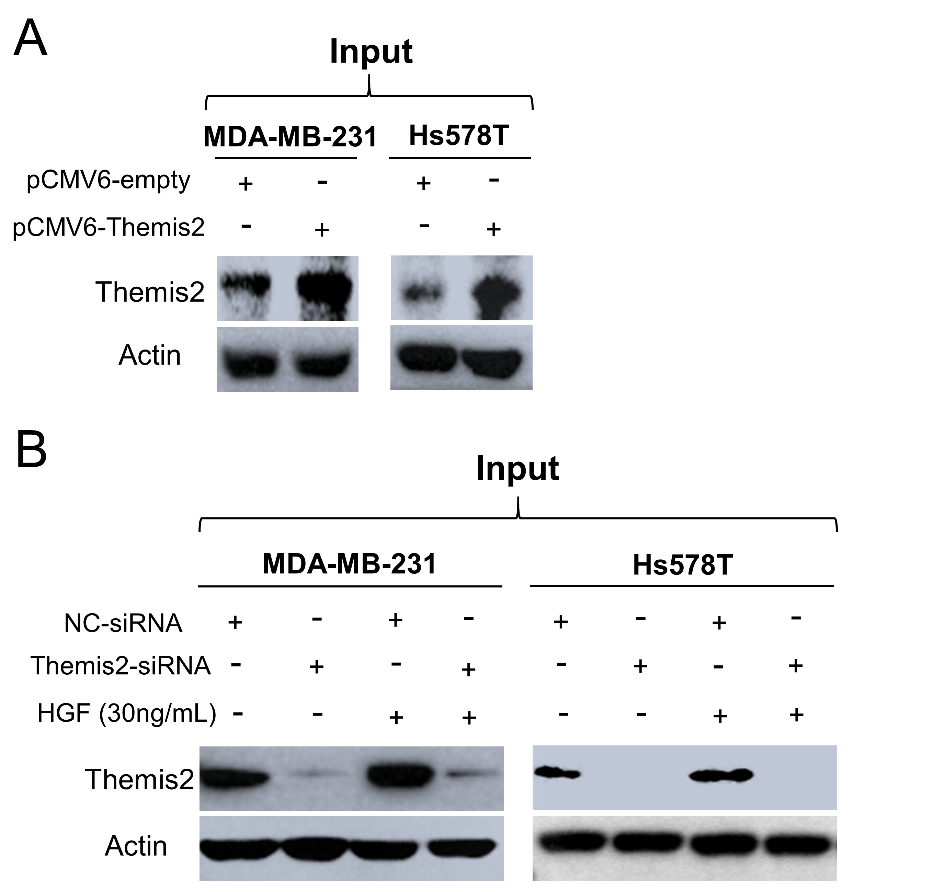


**Supplementary Figure. 13**


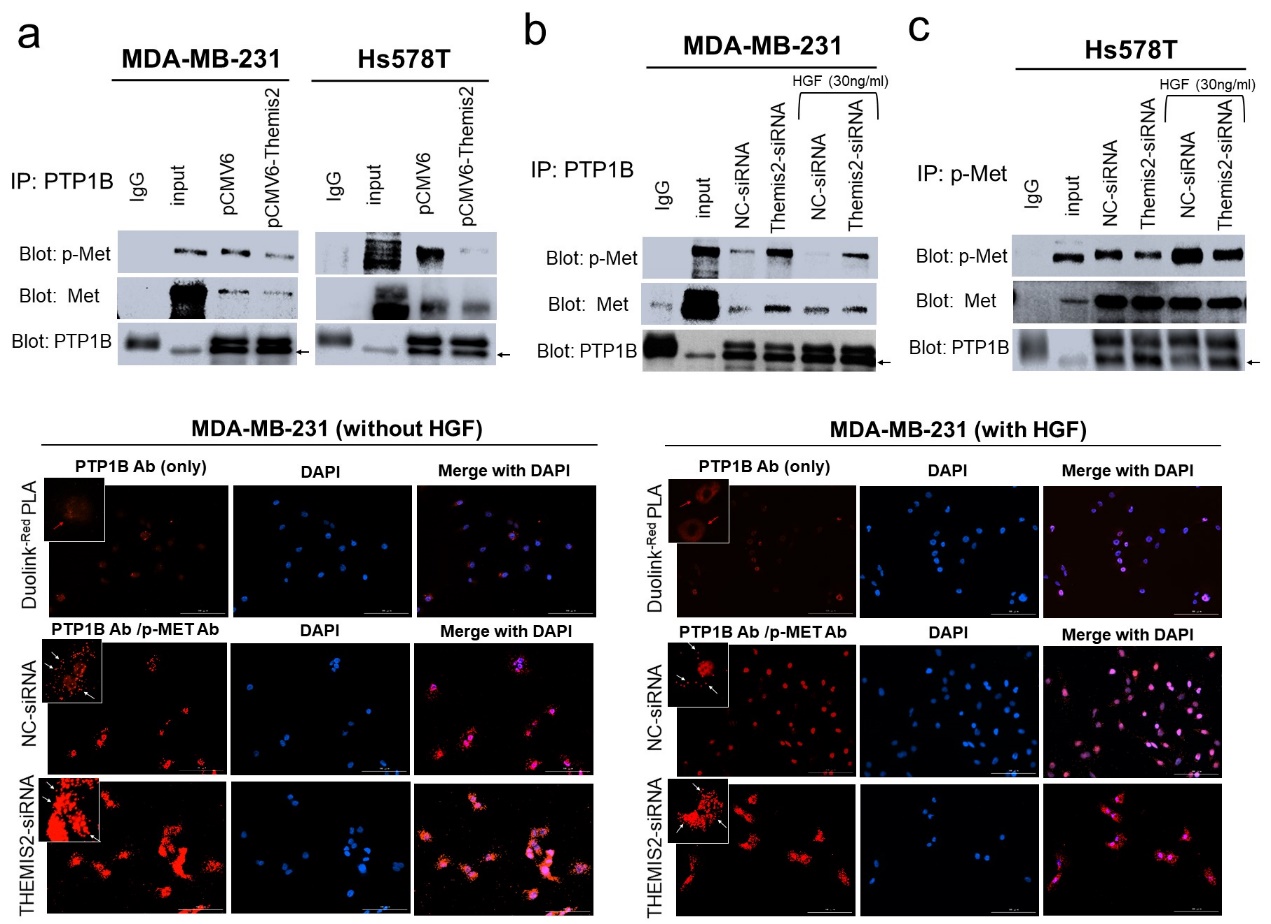


**Supplementary Figure. 14**


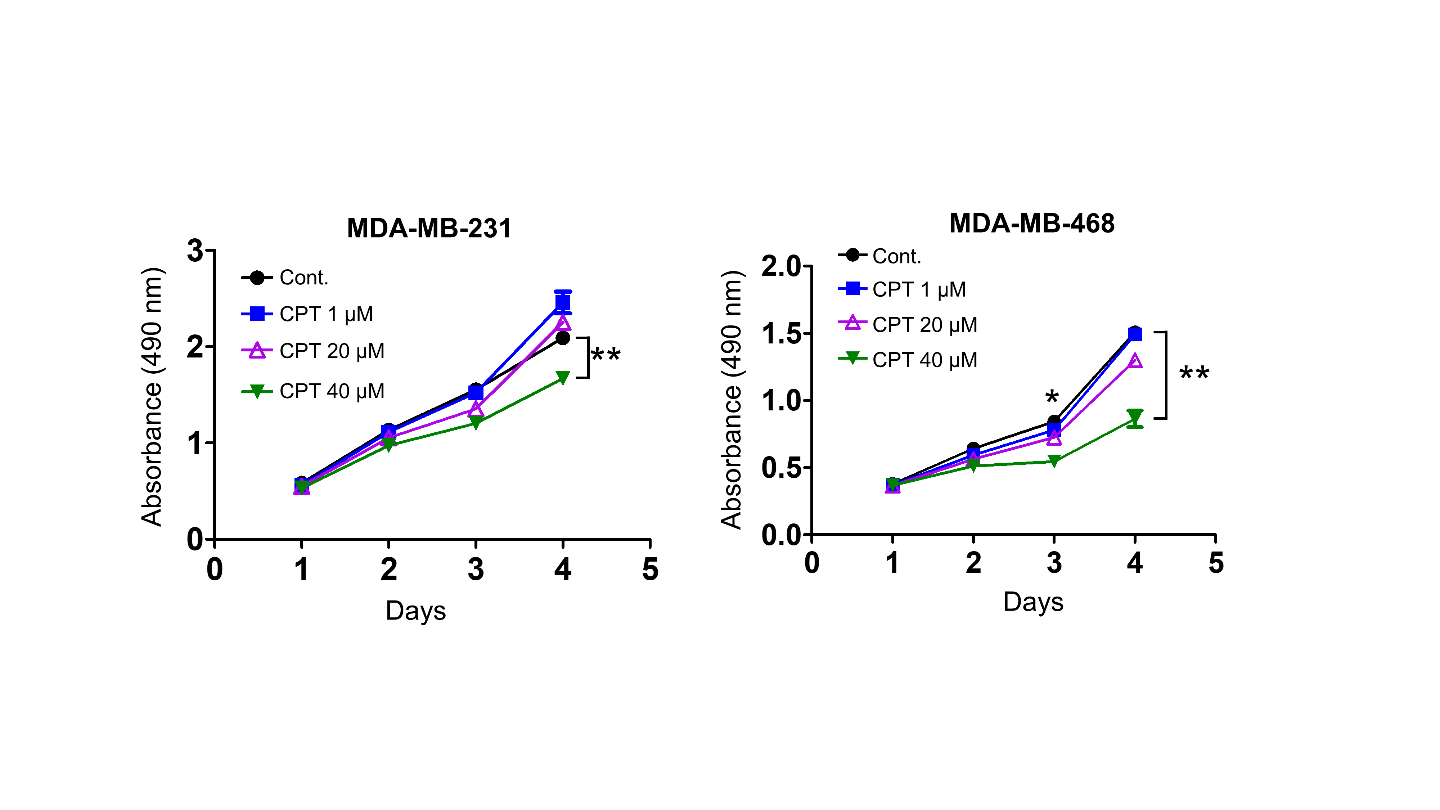


**Supplementary Figure. 15**


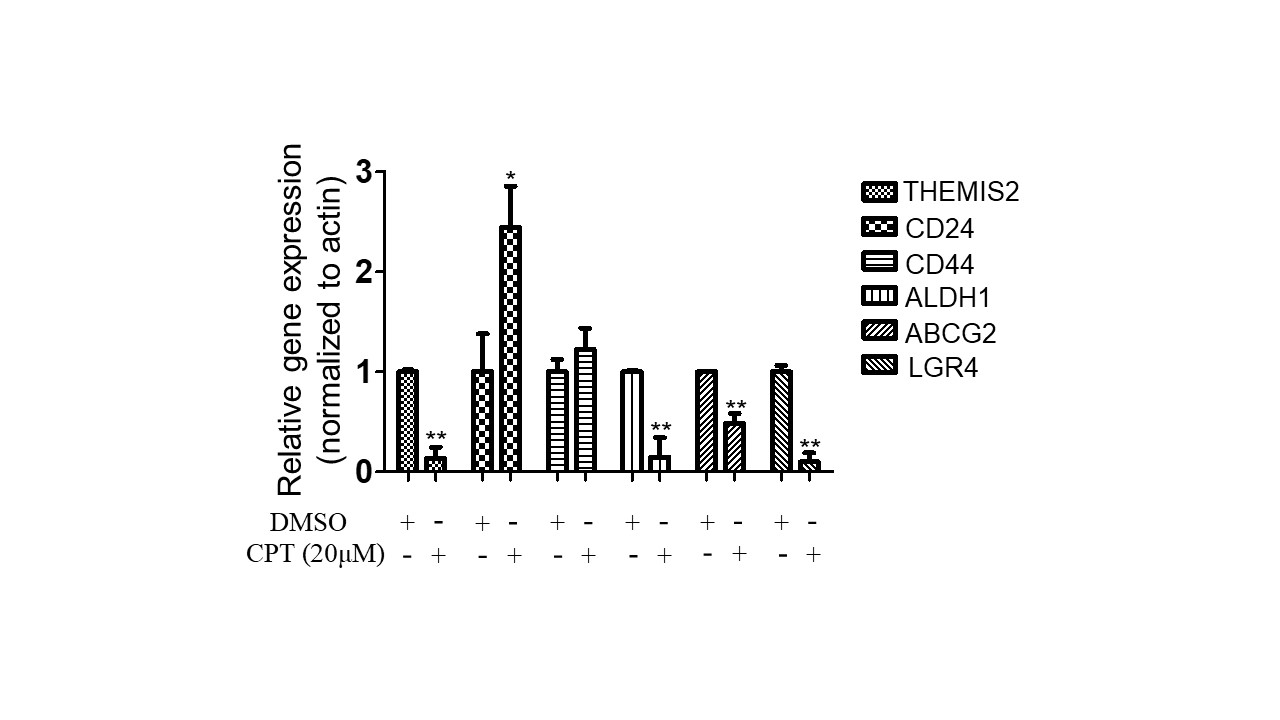


**Supplementary Figure. 16**


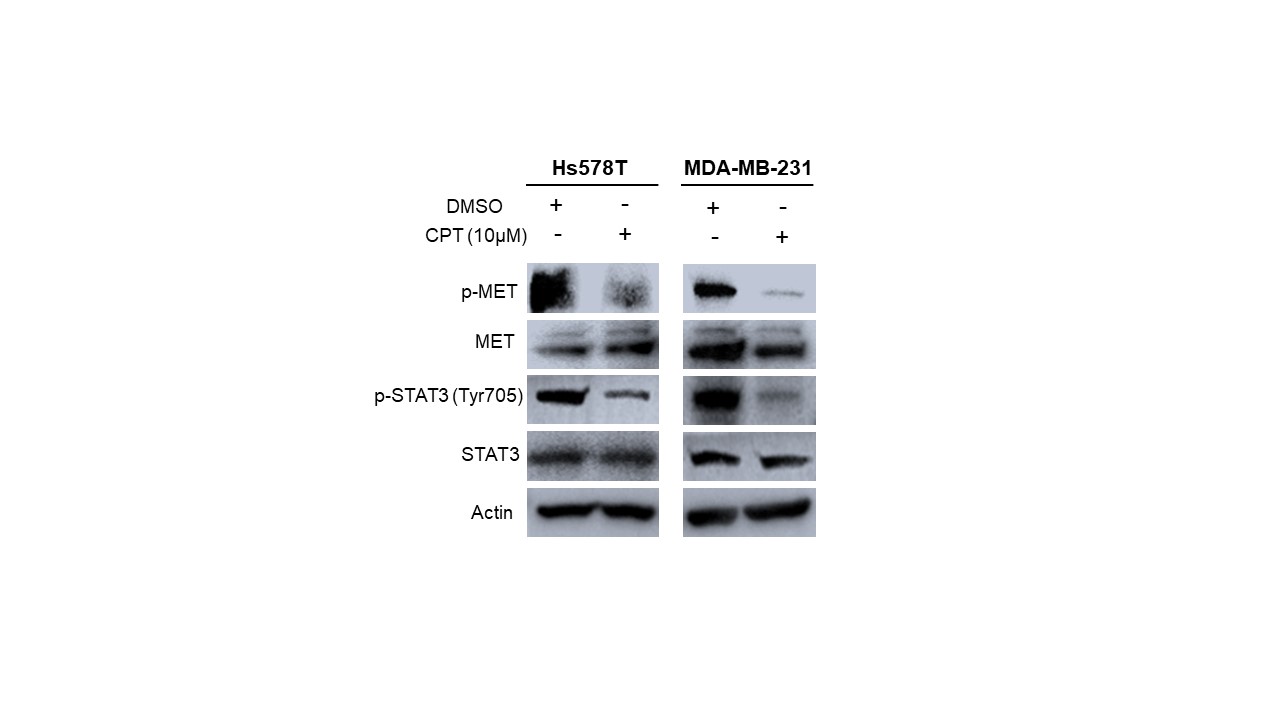


**Supplementary Figure. 17**


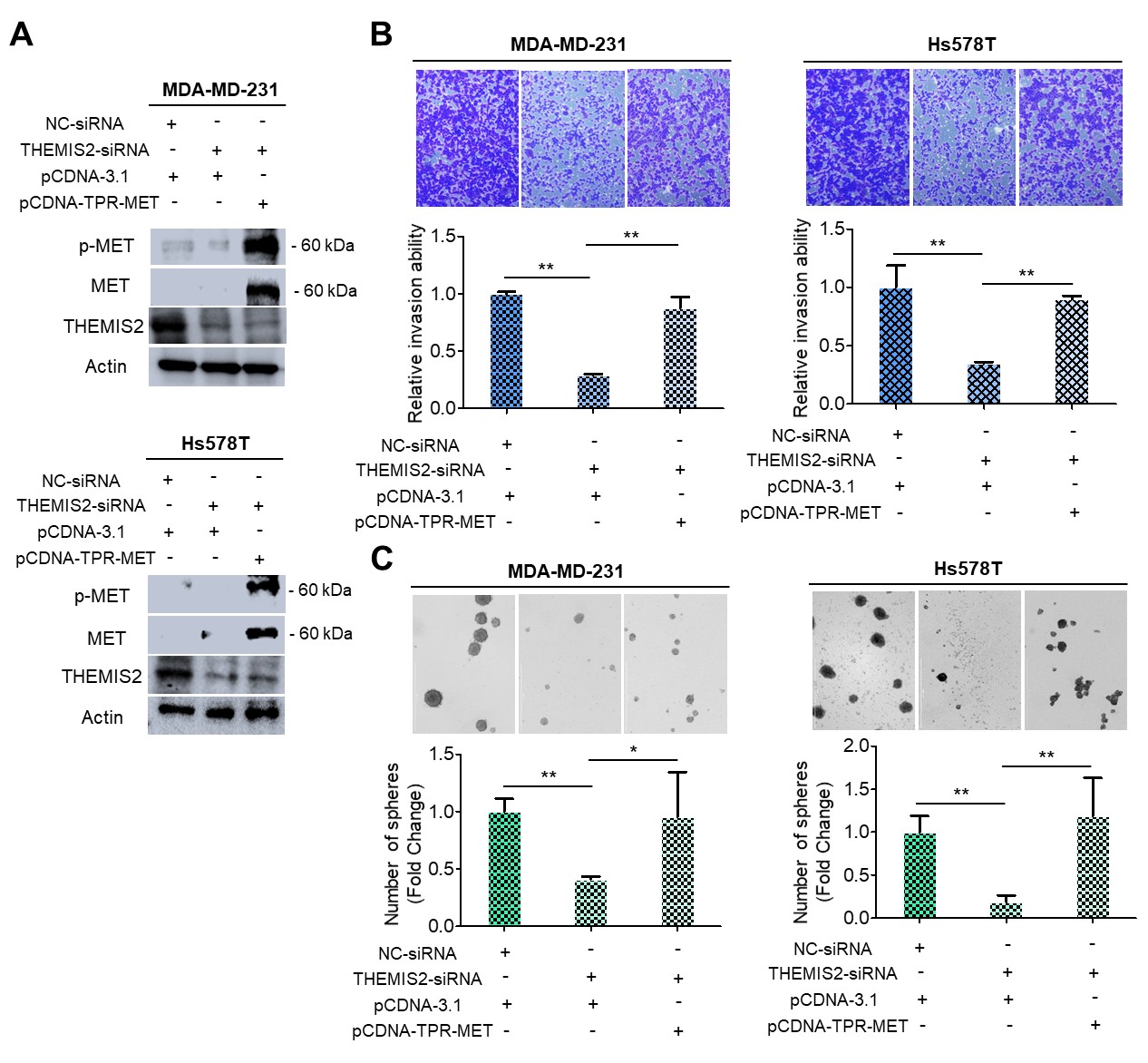


**Supplementary Figure. 18**

**
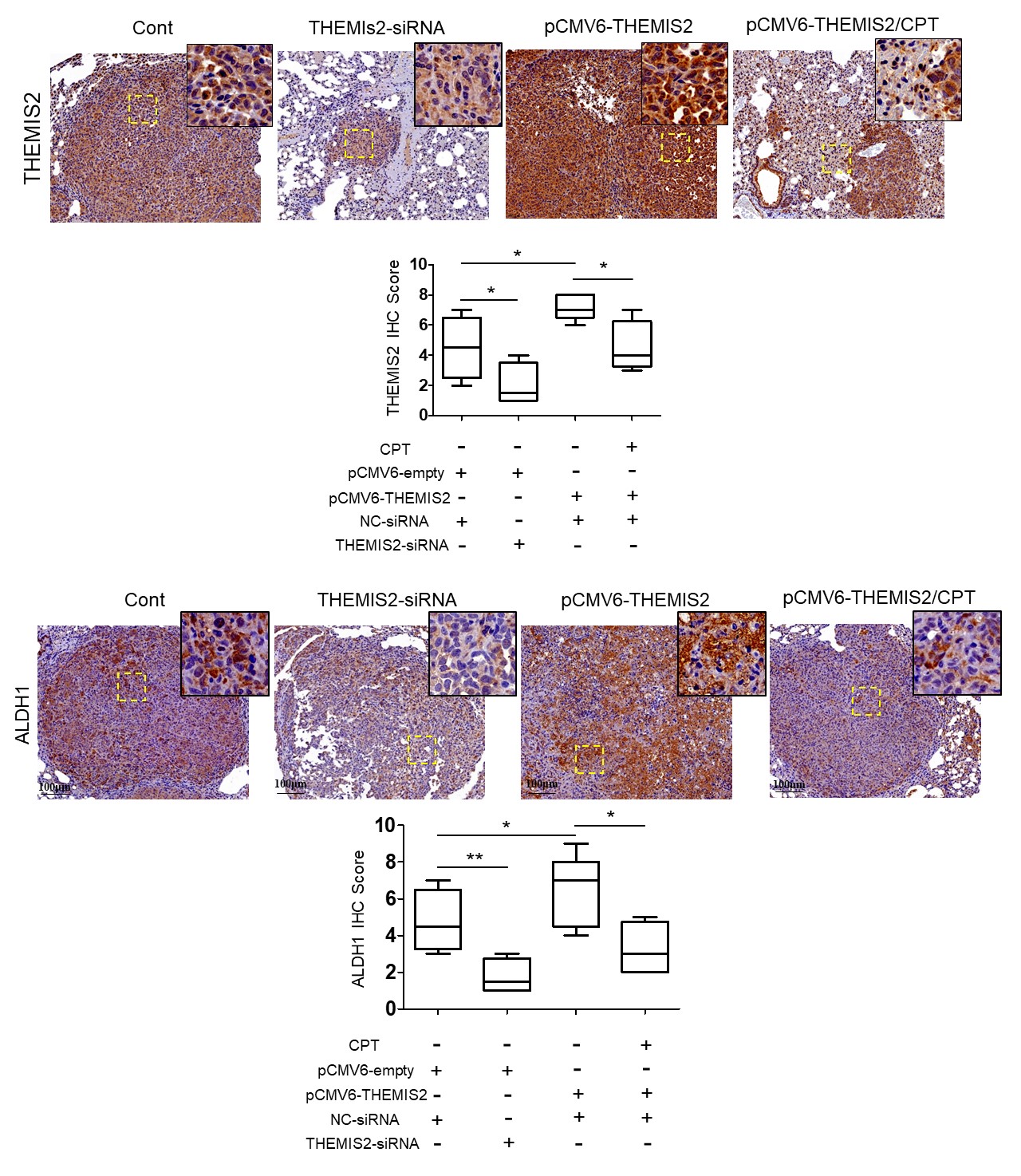
**

**Supplementary Figure. 19**

**
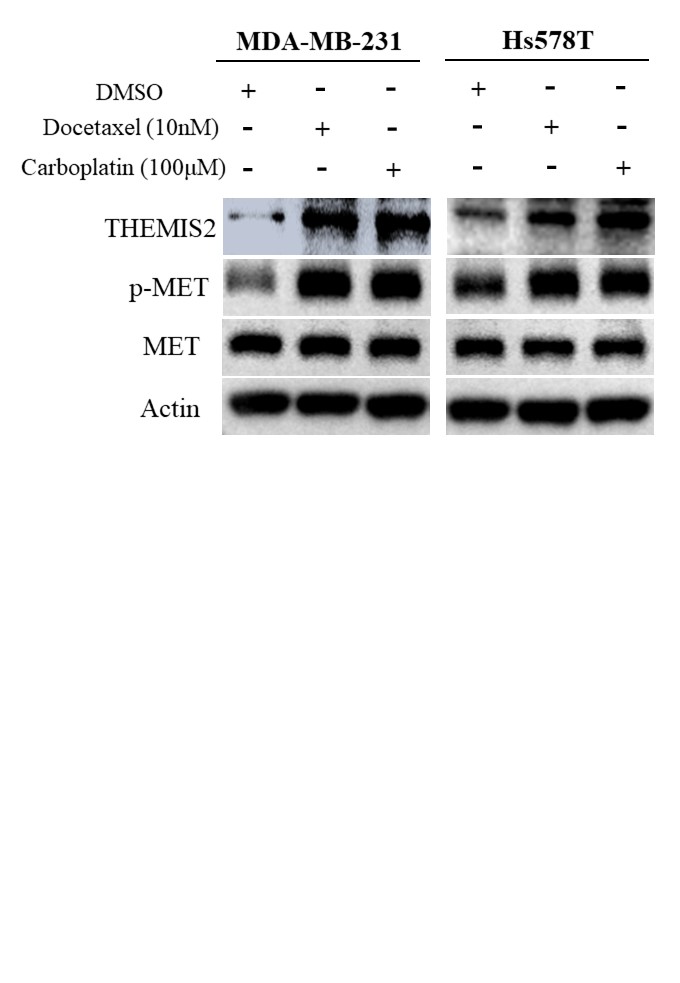
**

**Supplementary Figure. 20**

**
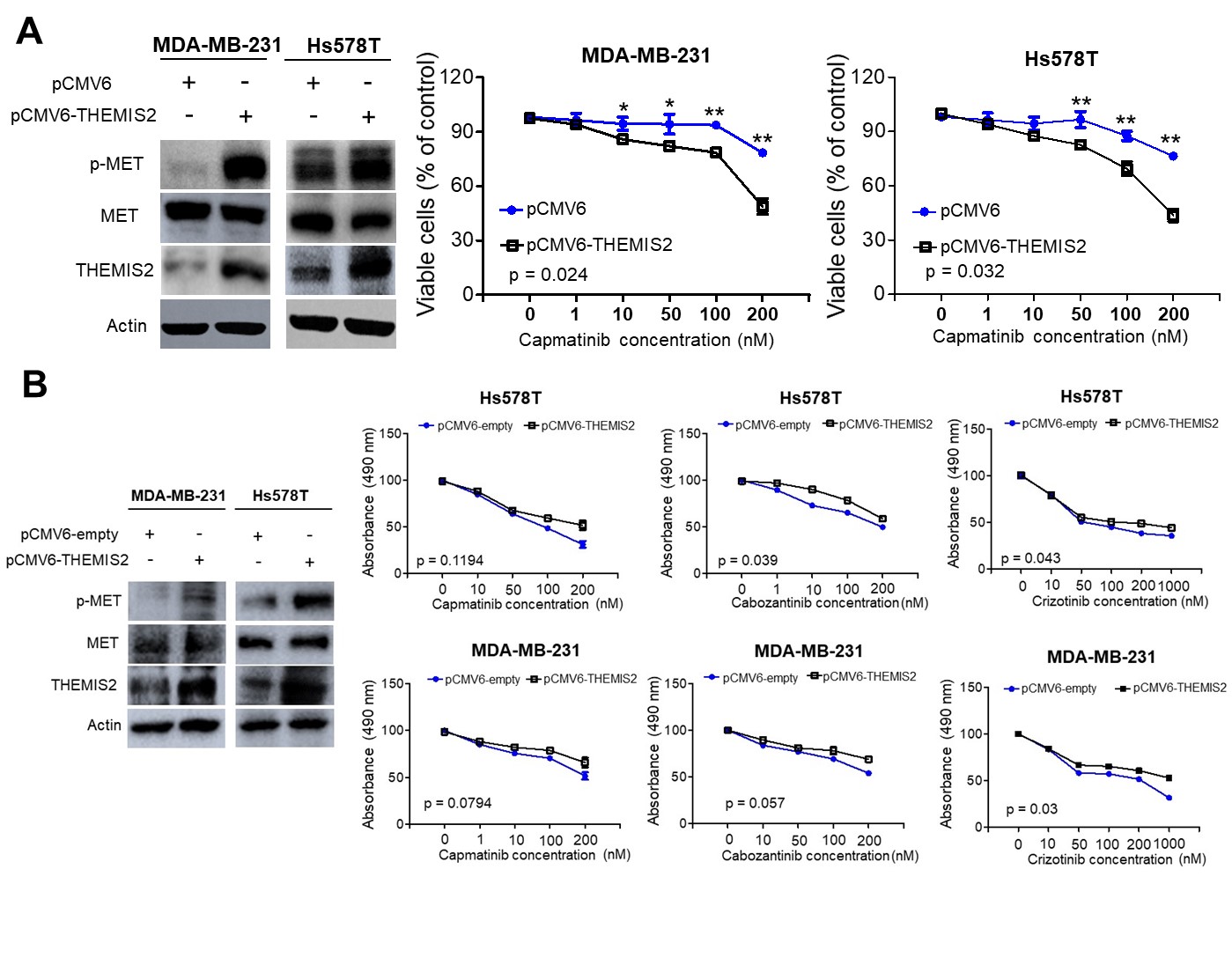
**

**Supplementary Figure. 21**


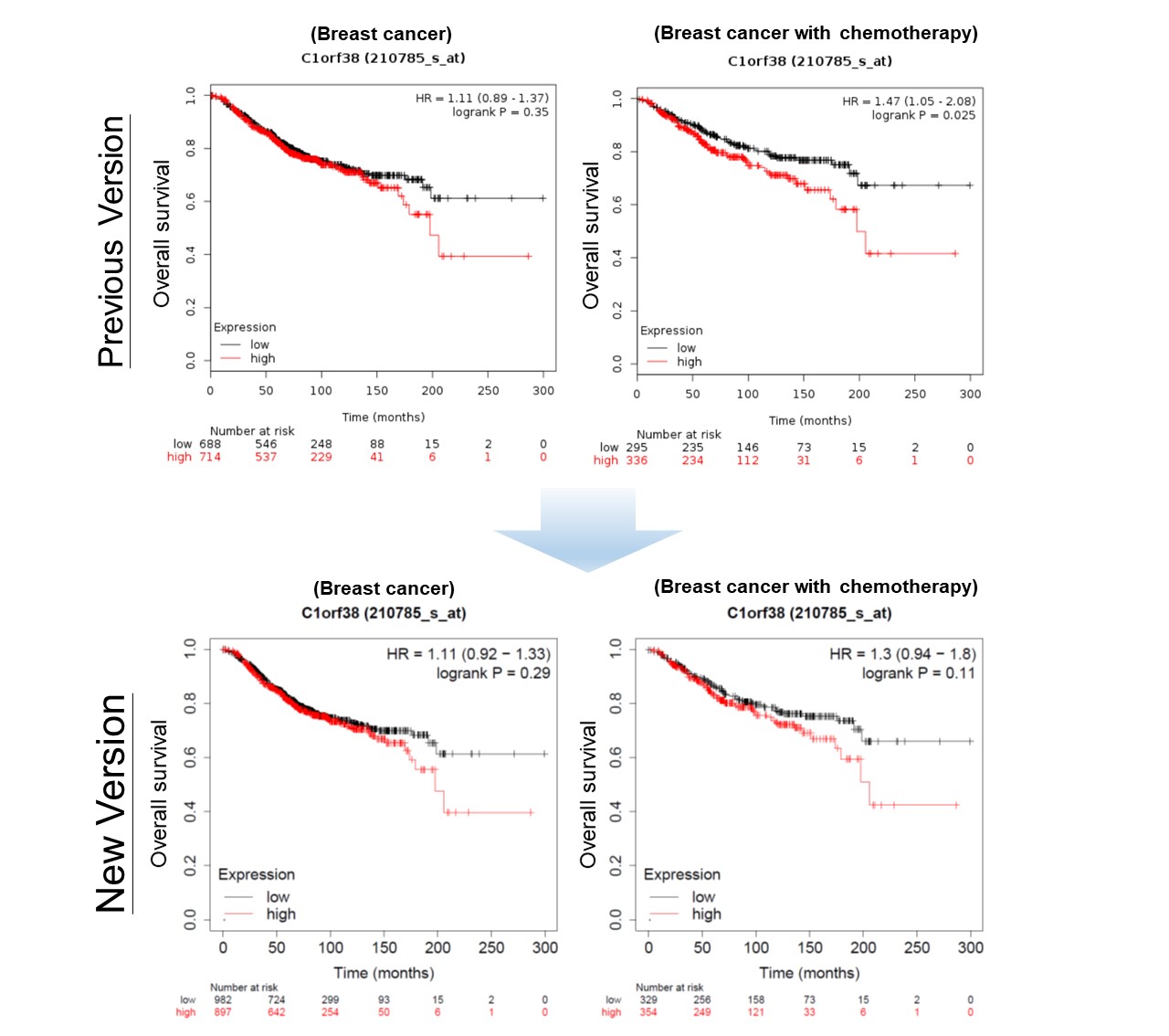


**Supplementary Figure. 22**


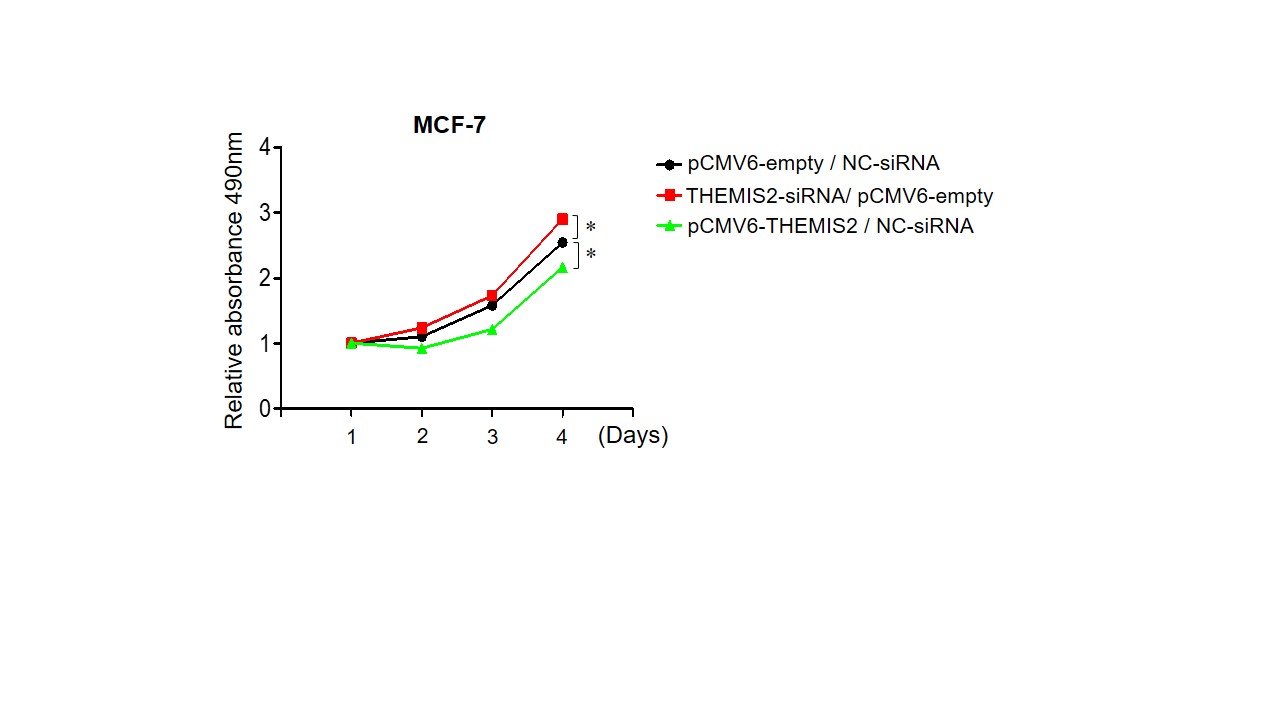

Supplement: Supplementary file 1 — Supplementary Figures [file 41388_2021_2136_MOESM1_ESM.docx]
