# Supplementary material for: Novel function of THEMIS2 in the enhancement of cancer stemness and chemoresistance by releasing PTP1B from MET
Source: Oncogene. 2022 Jan 1;41(7):997–1010. doi: 10.1038/s41388-021-02136-2 (PMC8837547; doi:10.1038/s41388-021-02136-2)
Supplement: Supplementary file 2 — Supplementary Figure legends [file 41388_2021_2136_MOESM2_ESM.docx]

**Supplementary Figure legends**

**Supplementary Figure. 1:** **Effect of knockdown or overexpression of THEMIS2 on the expression levels of CSC markers in TNBC**

**A** The protein expression levels of CSC markers in THEMIS2-depleted MDA-MB-231 and Hs578T cells, relative to those of their parental cells, were analyzed using immunoblotting. Actin was used as an internal control. **B** Breast CSC markers CD133 (red dots) and ALDH1 (green dots) on the surfaces of spheres in parental MDA-MB-231 and stable THEMIS2-overexpressing MDA-MB-231 (231) cells shown in confocal immunofluorescence images. The nuclei were stained with DAPI.

**Supplementary Figure. 2: The up- and down-regulation of THEMIS2 regulated cancer stemness and migration**

**A** The sphere-forming abilities of two stable THEMIS2-depleted TNBC lines (MDA-MB-231 (231) and Hs578T) were compared with those of their parental cells. The histograms represent means ± SEs from three independent experiments (*, *P* < 0.05; **, *P* < 0.01; ***, *P* < 0.001). **B** The migration abilities of three stable THEMIS2-expressing TNBC lines (Hs578T, MDA-MB-231, and BT549), relative to those of their respective parental cells, were analyzed using a chemotactic cell migration assay. The histograms represent means ± SEs from three independent experiments (*, *P* < 0.05; **, *P* < 0.01; ***, *P* < 0.001).

**Supplementary Figure. 3: THEMSI2 regulated cell proliferation and sphere formation in MDA-MB-231**

**A** The expression level of THEMIS2 regulated cell proliferation in MDA-MB-231 stable cells transfected with the indicated plasmids. MTT assay was conducted to measure cell growth after 96 h.  **B** The sphere-forming abilities of the stable THEMIS2-overexpressed or depleted MDA-MB-231 cells were compared with those of their parental cells. The histograms represent means ± SEs from three independent experiments (*, *P* < 0.05; **, *P* < 0.01; ***, *P* < 0.001).

**Supplementary Figure. 4: THEMIS2 regulated MET signaling molecules in TNBC cells with or without HGF treatment**

**A** Protein expression levels of RTKs in THEMIS2 siRNA–transfected MDA-MB-231 (231) and Hs578T cells were analyzed through immunoblotting. Actin was used as the internal control. **B** Through immunoblotting, the protein expression levels of p-MET, MET, and THEMIS2 in Hs578T cells transfected with THEMIS2 siRNA or controls, with or without HGF stimulation (30 ng/mL), were analyzed. Actin was used as the internal control. **C** The protein expression levels of p-MET, MET, and THEMIS2 in Hs578T cells transfected with pCMV6-THEMIS2 vector or controls and with or without HGF (30 ng/mL) stimulation were analyzed through immunoblotting. Actin was used as the internal control. **D** Negative control siRNA and THEMIS2-silenced MDA-MB-231 cells were serum starved for 18 h, treated with 30 ng/mL HGF for 30 min, and then subjected to immunoblotting. Knockdown of THEMIS2 led to a reduction in the HGF-induced phosphorylation of MET, as well as that of its downstream signaling mediators.

**Supplementary Figure. 5: The association betweenTHEMIS2 and MET were elevated in sphere cells compared to parental cells.**

**A** The association of THEMIS2 and MET in spheres, which were strengthened in HS578T sphere cells by Duolink PLA assay. The nuclei were stained with DAPI. **B** To obtain separated Hs578T sphere cells, we digested the spheres by trypsin and seeded on slide for O/N. A Duolink PLA was then performed. Top i: MET antibody only served as the negative control; ii: protein–protein interactions between MET and THEMIS2 using both antibodies in parental cells; iii: protein–protein interactions between MET and THEMIS2 in sphere cells. Nuclei were counterstained with DAPI (blue). Bottom: Quantification of signal by number of PLA puncta per cell. C TNBC cells and its sphere cells were used in the co-IP experiments. Cell lysates were blotted directly or subjected to IP with a MET or THEMIS2 antibody; this was followed by blotting with the indicated antibodies. IgG served as the negative control.

**Supplementary Figure. 6:** Ectopic expression of THEMIS2 partially rescued the Capmatinib-mediated inhibition of sphere formation

**A** Protein expression levels of p-MET, MET, CD133, and ALDH1 in MDA-MB-231and Hs578T cells transfected with pCMV6-THEMIS2 vector or control, as well as cell lysates harvested 24 h after treatment with the MET inhibitor Capmatinib were analyzed through immunoblotting. Actin was used as the internal control. **B** The sphere-forming abilities of stable THEMIS2-overexpressing MDA-MB-231cells, compared with those of their parental cells. The inhibition of these abilities after treatment with Capmatinib (10 μM) was rescued by the ectopic expression of THEMIS2. The histograms represent means ± SEs from three independent experiments (*, *P* < 0.05; **, *P* < 0.01).

**Supplementary Figure. 7:** Overexpression of THEMIS2-mediated MET activation and increased expression of the CSC markers, CD133 and ALDH1, in MDA-MB-231 and Hs578T cells were suppressed by Capmatinib (100nM).

**Supplementary Figure. 8: Immunoblotting analysis of p-MET in the indicated PTP siRNA–transfected MDA-MB-231 cells.**

**Supplementary Figure. 9: Depletion of PTP1B reversed the THEMIS2 depletion–mediated inhibition of MET phosphorylation.**

MDA-MB-231 cells were transfected with the indicated siRNA for 8 h, serum starved for 24 h, and then treated with 30 ng/mL HGF. The protein expression levels of MET and p-MET in the indicated siRNA transfected MDA-MB-231 cells were analyzed through immunoblotting. Actin was used as the internal control.

**Supplementary Figure. 10:** Overexpression of THEMIS2 reduced the association of PTP1B with MET or with p-MET

MDA-MB-231, Hs578T, MDA-MB-468 and BT549 cells were used in the co-IP experiments. Cell lysates were blotted directly or subjected to IP with a PTP1B antibody first; this was followed by blotting with the indicated antibodies. IgG served as the negative control.

**Supplementary Figure. 11:** PTP1B-MET interaction was decreased in THEMIS2 overexpressing MDA-MB-231 cells.

**A** MDA-MB-231 cells were transfected with the pCMV6-empty or pCMV6-THEMIS2. The Duolink PLA was then performed. Top: p-MET antibody only served as the negative control; middle: protein–protein interactions between MET and PTP1B using both antibodies; bottom: protein–protein interactions between p-MET and PTP1B in THEMIS2 overexpressing cells. The signals are represented as white arrows and the nonspecific signal as red arrows. Representative images of PLA (white arrow) and quantification of PLA (B) are shown. The histograms represent means ± SEs from three independent experiments (**, *P* < 0.01).

**Supplementary Figure. 12: Immunoblotting revealed protein expression levels of THEMIS2 under knockdown or overexpression.**

**A** Protein expression levels as reflected by the immunoblotting of THEMIS2 in MDA-MB-231 and Hs578T cells transfected with control or a THEMIS2 plasmid. **B** MDA-MB-231 and Hs578T cells were transfected with the THEMIS2 siRNA for 8 h, serum starved for 24 h, and then treated with 30 ng/mL HGF. The THEMIS2 protein expression level was determined through immunoblotting. Actin was used as the internal control.

**Supplementary Figure. 13:** PTP1B-MET interaction was increased in THEMIS2-depleted MDA-MB-231 cells with or without HGF.

MDA-MB-231 cells were transfected with the negative control siRNA or THEMIS2 siRNA for 8 h, serum starved for 24 h, and then treated without or with 30 ng/mL HGF for 30 min (left and right panels, respectively). A Duolink PLA was then performed. Top: PTP1B antibody served as the negative control; middle: protein–protein interactions between PTP1B and p-MET using both antibodies; bottom: protein–protein interactions between PTP1B and p-MET in THEMIS2-depleted cells. The close proximity (<40 nm) of the two proteins in each set is indicated by small, distinct red dots, which were detected using fluorescence microscopy. The signals are represented as white arrows and the nonspecific signal as red arrows. Nuclei were counterstained with DAPI (blue).

**Supplementary Figure. 14: Effects on cell proliferation were assessed through an MTT assay in MDA-MB-231 and MDA-MB-468 cells after treatment with various concentrations (1, 20, and 40 μM) of CPT for 24, 48, 72, and 96 h.** The points represent means ± SEs from three independent experiments (*, *P* < 0.05; **, *P* < 0.01).

**Supplementary Figure. 15: As determined through qRT-PCR, treatment with 20 μM CPT led to reduced mRNA expression levels of THEMIS2, ALDH1, ABCG2, and LGR4, as well as an increase in the mRNA expression level of CD24.** The histograms represent means ± SEs from three independent experiments (*, *P* < 0.05; **, *P* < 0.01).

**Supplementary Figure. 16: Treatment with CPT decreased MET–STAT3 signaling in TNBC cells.**

Protein expression levels of p-MET, MET, p-STAT3 (Tyr705) and STAT3 in Hs578T and MDA-MB-231 cells were treated with CPT (10 μM) for 24 h and analyzed by immunoblotting. Actin was used as the internal control.

**Supplementary Figure. 17: Ectopic expression of TPR-MET reversed the THEMIS2-depletion mediated inhibition of invasion and sphere formation in TNBC cells.**

**A** The protein levels of p-MET, MET, and THEMIS2 in MDA-MB-231 and Hs578T cells transfected with control, THEMIS2 siRNA or THEMIS2 with or without pCDNA-TPR-MET were analyzed through immunoblotting. Actin was used as the internal control. **B** The invasion abilities were reversed in THEMIS2-depleted cells by ectopic expression of pCDNA-TPR-MET. C Sphere formation under stem cell selective condition was examined on day 4~8 after culturing of the cells transfected with the indicated siRNA and plasmids.

**Supplementary Figure. 18: Representative examples of the expression levels of THEMIS2 and ALDH1 protein as determined through IHC staining in the four indicated mice groups.**

**Supplementary Figure. 19: Correlation among the expression of THEMIS2 and MET phosphorylation.**

THEMIS2 protein expression levels in MDA-MB-231 and Hs578T cells with or without treatment with docetaxel (10 nM) or carboplatin (100 μM) for 1 week.

**Supplementary Figure. 20:** The effects of upregulated THEMIS2 on drug resistance in stable or transiently transfected TNBC cells.

**A** Dose-dependent growth inhibition of MDA-MB-231 and Hs578T stable cells under continuous exposure to the indicated concentrations of Capmatinib for 24 h was measured using an MTT assay. **B** The THEMIS2 transiently transfected TNBC cells did not display increased sensitivity toward the three MET inhibitors (Capmatinib, Cabozantinib and Crizotinib).

**Supplementary Figure. 21:** Kaplan-Meier analysis of the THEMIS2 expression in breast cancer patients and those received chemotherapy. (https://kmplot.com/)

The upper panel was original from 01 January 2020 (210785_s_at database) and lower panel was original form 01 August 2021 (210785_s_at database). The association of THEMIS2 expression levels with overall survival in all patients with breast cancer, in patients who received chemotherapy.

**Supplementary Figure. 22: THEMSI2 regulated cell proliferation in MCF-7**

The expression level of THEMIS2 regulated cell proliferation in MCF-7 cells transfected with the indicated plasmids. MTT assay was conducted to measure cell growth after 96 h.
