# Supplementary material for: Novel function of THEMIS2 in the enhancement of cancer stemness and chemoresistance by releasing PTP1B from MET
Source: Oncogene. 2022 Jan 1;41(7):997–1010. doi: 10.1038/s41388-021-02136-2 (PMC8837547; doi:10.1038/s41388-021-02136-2)
Supplement: Supplementary file 3 — Supplementary Table 1 [file 41388_2021_2136_MOESM3_ESM.docx]

**Supplementary Table 1. Primers and siRNAs used in this study.**

| Oligonucleotide sequences used for qRT-PCR |
| --- |
| Gene Forward primer (5’-3’) Reverse primer (5’-3’) |
| **THEMIS2** TGTAGCACCAAGCCTGATAG CCAAGGTCCTAAGGTTGTGT  **CD24** TGCTCCTACCCACGCAGATT GGCCAACCCAGAGTTGGAA  **CD44** AAGGGCACGTGGTGATTCC GGTCTGTGACTGATGTACAATCTT  **ALDH1**  ACCATTCATTGACTCCATTGTCG ACAAGTTGGCTGATTTAATCGAA  **ABCG2** CCTACAACTGGCTTAGACTC GAGGCTGATGAATGGAGAAG  **LGR4** GGAGCATTTGATGGTAATCCACTC CCATGCTTGCACCACGAATGAC  **Actin**  CGGCATCGTCACCAACTG TCTCAAACATGATCTGGGTCATCT |

| The sequences of genes siRNA oligonucleotides |
| --- |
| **Gene**  **Sequences of siRNA oligonucleotides** |
| **THEMIS2 SMART-pool:**  1. CCAUCUAAGUGCUGGAGGAtt  2. CCAAGAUUAUUUCAUUUUAtt  3. GAGAGGUCCCUCCCAUCU Utt  **PTP1B siRNA:**   1. GGAUUAAACUACAUCAAGAtt 2. GGAGAAAGGUUCGUUAAAAtt   **SHP1 siRNA:**   1. ACCUCUCCCUGACCCUGUAtt 2. CCCAUAUUCGGAUCCAGAAtt   **SHP2 siRNA:**   1. GGAUGGUGUUCCAAGAAAAtt 2. CCAAUGAGCCUGUUUCAGAtt |
