# Supplementary material for: Novel function of THEMIS2 in the enhancement of cancer stemness and chemoresistance by releasing PTP1B from MET
Source: Oncogene. 2022 Jan 1;41(7):997–1010. doi: 10.1038/s41388-021-02136-2 (PMC8837547; doi:10.1038/s41388-021-02136-2)
Supplement: Supplementary file 4 — Supplementary Materials [file 41388_2021_2136_MOESM4_ESM.docx]

Supplementary materials

**Web server survival analysis**

The analysis of patient survival related to the expression of specific genes was performed on the pan-cancer RNA-sequencing (gene-chip) web server. Kaplan-Meier plots were generated through the automatic selection of the optimal cutoff values between lower and upper quartiles, which formed the high and low expression groups. This analysis was performed on patients with breast cancer who had either received effective chemotherapy treatment or no treatment (<https://kmplot.com/analysis/>). Clustering of the transcriptomic profiles of 78 triple-negative breast cancer (TNBC) cases was obtained from the ONCOMINE database.

**Cell culture and sphere forming assay**

Human TNBC cell lines (MDA-MB-231, MDA-MB-231-IV2, MDA-MB-468, BT549 and Hs578T)(Chan *et al*, 2014), and human ovarian cancer cell lines (SKOV-I6 and OVS1) were used(Tung *et al*, 2017) described previously. The Hs578T line was selected for one cycle through the orthotopic injection of breast cancer cells into the mammary fat pads of CB17 severe-combined immunodeficient (CB17-SCID) mice. Next, the tumor cells grown from the mammary fat pads were isolated to obtain Hs578T-FPI (Hs578T-Fat-Pad-Invasive). In brief, cells were grown in Dulbecco’s Modified Eagle Medium (DMEM; Invitrogen, Carlsbad, CA, USA) with 10% fetal bovine serum (FBS; Invitrogen) and incubated at 37°C in 5% CO_2_. Breast cancer and ovarian cancer cells were cultured in a stem cell selective condition to allow sphere formation, as described previously (Tung *et al.*, 2017).

**Cell proliferation assay**

To examine whether CPT could inhibit cell proliferation in TNBC, MDA-MB-231 cells were treated with different doses (1, 20 and 40 μM) of CPT for 4 days. Other procedures were performed as described previously (Tung *et al.*, 2017).

**Transfection of indicated small interfering RNA and the expression vector**

Non-specific and human THEMIS2 SMART-pool-targeting small interfering RNA molecules were obtained from Dharmacon. PTP1B, SHP1, and SHP2 siRNAs were purchased from Invitrogen (Table S1). The siRNAs were transfected into selected cells for 24 h, using a specialized reagent (Santa Cruz Biotechnology; category number sc-29528) according to the manufacturer’s protocol. The full-length THEMIS2 cDNA was inserted into the pCMV6-control plasmid. Both MDA-MB-231 and Hs578T cells were transfected with pCMV6-puro-THEMIS2, and stable THEMIS2-expressing cancer cell lines were selected by using puromycin (Sigma-Aldrich). Cells were transfected using Lipofectamine 2000 and Lipofectamine RNAiMAX (Invitrogen).

**Co-immunoprecipitation assay and immunoblotting analysis**

MDA-MB-231-IV2 cell extracts containing 1 mg of protein were immunoprecipitated for 2 h at 4°C through incubation with the antibodies for MET or THEMIS2, respectively. The immune complexes were captured with protein A or protein G agarose through overnight incubation. The agarose beads containing antibody-protein complexes were collected by centrifugation and washed three times with lysis buffer. Protein concentrations were determined using the Bradford method (Bio-Rad, Hercules, CA, USA). The cell lysates (30μg per lane) were separated using 10% SDS-PAGE and transblotted onto a polyvinylidene difluoride (PVDF) membrane (Millipore, Bedford, MA, USA). The membrane was incubated at room temperature in a blocking solution of phosphate-buffered saline (PBS) containing 5% powdered skim milk and 0.02% sodium azide for 1 h. This was followed incubation for 2 h in a solution containing an appropriate dilution (1:1000) of primary antibody (e.g., anti-THEMIS2, anti-CD44, anti-CD133, anti-Nanog, anti-OCT4, anti-Snail, anti-Slug or anti-β-actin). After the membrane was washed three times, it was incubated in PBS containing horseradish peroxidase conjugated goat anti-mouse immunoglobulin (IgG) (1:5000; Sigma, St. Louis, MO, USA) for 1 h. The membrane was then washed three times with PBS and signals were developed with chemiluminescence reagent (Amersham Pharmacia Biotech, Little Chal-font, UK).

**Cell chemotactic migration and invasion assay**

Control or specific plasmid transfected cells (5 × 10^4^) were harvested from monolayer culture, resuspended in serum-free medium with 0.1% bovine serum albumin (Sigma-Aldrich), and then plated onto a Transwell Boyden chamber, with or without a Matrigel coating on the upper chamber, for invasion and migration assays respectively. The chambers were incubated in DMEM for 24h with the addition of 10% FBS to the lower chamber. Cells that did not move were removed using cotton swabs, and the chambers were stained with crystal violet. Photomicrographs of three random regions were captured from duplicated assay chambers. The cell numbers were counted and normalized to the control. All experiments were repeated three times.

**Soft agar assay**

The base agar, consisting of 1 mL of the preheated DMEM mixed with 10% FBS containing 0.5% agarose, was loaded into a 12-well plate and then incubated at 37°C to solidify. Next, 5 × 10^3^ cells were quickly mixed with 1 mL of the preheated DMEM with 10% FBS containing 0.25% melted agarose (not exceeding 40°C to maintain cell vitality) and loaded above the base agar to form a top layer. After the cell/agar mixture had solidified at room temperature for 30 min, the plate was incubated at 37°C for 14 days. Colonies were stained with 0.05% (wt/vol) iodonitrotetrazolium chloride (Sigma) for 2 days. The colonies were photographed and quantified using the ImageJ software packsge (NIH, USA).

**Immunofluorescence microscopy**

Cells were washed with PBS and fixed with 4% formaldehyde for 5 min, washed three times with PBS, treated with 0.1% Triton for 10 min, and blocked with 5% goat serum for 1 h. Subsequently, they were incubated with THEMIS2 antibody at 200× dilution overnight at 4°C. This was followed by Alexa Fluor 488 goat anti-rabbit IgG for green fluorescence incubation with MET antibody (200× dilution) at 4°C overnight, and binding with Rhodamine Red-X goat anti-rabbit IgG for red fluorescence.

**Duolink proximity ligation assay**

*In situ* proximity ligation assays (PLAs) of the fixed cells were performed according to the manufacturer’s protocol (category number DUO92008, Sigma-Aldrich). In brief, coverslips were blocked for 45 min at 37°C with a blocking solution and then incubated with two primary antibodies (THEMIS2/MET or PTP1B/p-MET antibodies) at 1:200 dilution overnight at 4°C. The two primary antibodies had to be generated from different species (i.e., mouse/rabbit, rabbit/goat, or mouse/goat). Next, the coverslips were washed for 10 min three times with PBS containing 0.1% Tween 20 under gentle shaking. The cells were then stained with secondary antibodies (2-Ab) known as PLA probes (one PLUS and one MINUS, Sigma-Aldrich) for 2 h at 37°C. Next, the coverslips were washed for 10 min three times in buffer B (DUO82048, Sigma-Aldrich) under gentle shaking and then incubated with a buffer containing DNA ligase (1 unit/μL) for 30 min at 37°C. Subsequently, they were washed for 10 min three times in buffer B under gentle shaking and then incubated with an amplification buffer containing DNA polymerase (10 unit/Μl; Cat. No. DUO82030, Sigma-Aldrich) for 2 h at 37°C. Finally, the coverslips were rinsed with buffer A (10 unit/μL; Cat. No. DUO82030, Sigma-Aldrich) for 10 min and then washed for 5 min with 0.1× buffer B. The dried coverslips were treated with mounting solution (P36981, Invitrogen) containing 4′, 6-diamidino-2-phenylindole (DAPI).

**Immunohistochemistry (IHC) scoring**

IHC staining was used to evaluate THEMIS2 protein expression in breast cancer tissue arrays. The THEMIS2 antibody (ab236975) was purchased from Abcam, Inc. The IHC score of THEMIS2 for each specimen were defined as the cell staining intensity (0=negative; 1=mild; 2=moderate; and 3=strong) multiplied by the percentage of labeled cells (0-100%), leading to scores from 0 to 300. A score higher than the mean was defined as ‘high’ expression, while a score equal to or lower than the mean was categorized as ‘low’ expression in tumor.

**Transcriptome sequencing**

The transcriptome sequencing experiments involved RNA extraction and quality control analysis, library construction, purification, and library quality control analysis and quantification, as well as sequencing cluster generation and high-throughput sequencing. To ensure the accuracy and reliability of the results, each step was subjected to strict monitoring and quality control. After the libraries were mixed on the basis of their effective concentration and the required sequencing data volume, high-throughput sequencing was performed by using the Illumina sequencing platform. The clean data were aligned to the reference genome as described previously(Kim *et al*, 2015).

**References**

Chan SH, Huang WC, Chang JW, Chang KJ, Kuo WH, Wang MY, Lin KY, Uen YH, Hou MF, Lin CM *et al* (2014) MicroRNA-149 targets GIT1 to suppress integrin signaling and breast cancer metastasis. *Oncogene* 33: 4496-4507

Kim D, Langmead B, Salzberg SL (2015) HISAT: a fast spliced aligner with low memory requirements. *Nat Methods* 12: 357-360

Tung SL, Huang WC, Hsu FC, Yang ZP, Jang TH, Chang JW, Chuang CM, Lai CR, Wang LH (2017) miRNA-34c-5p inhibits amphiregulin-induced ovarian cancer stemness and drug resistance via downregulation of the AREG-EGFR-ERK pathway. *Oncogenesis* 6: e326
